# Predictors of subjective and physiological craving during VR-cue exposure in alcohol use disorder: an explorative single-arm clinical study

**DOI:** 10.1186/s12888-026-08544-6

**Published:** 2026-08-26

**Authors:** Alva Lütt, Nadja Ruckser, Alessandro Turno, Nikolaos Tsamitros, Jil Zoé Fuhrmann, Daniel Schulze, Philipp Stepnicka, Daa Un Moon, Felix Bermpohl, Malek Bajbouj, Robert Schöneck, Miriam Sebold, Stefan Gutwinski, Anne Beck

**Affiliations:** 1 Department of Psychiatry and Neurosciences, Charité Campus Mitte, Charité – Universitätsmedizin Berlin, Berlin, Germany; 2 Department of Psychiatry and Psychotherapy, Charité at St. Hedwig Hospital, Charité – Universitätsmedizin Berlin, Berlin, Germany; 3 BIH Charité Junior Digital Clinician Scientist Program, Berlin Institute of Health at Charité – Universitätsmedizin Berlin, BIH Biomedical Innovation Academy, Charitéplatz 1, 10117 Berlin, Germany; 4 Partner Site Berlin-Potsdam, German Center for Mental Health (DZPG), Berlin, Germany; 5 Department of Education and Psychology, Clinical Psychological Intervention, Freie Universität Berlin, Berlin, Germany; 6 Department of Neurology with Experimental Neurology, Charité – Universitätsmedizin Berlin, Campus Benjamin Franklin, 12200 Berlin, Germany; 7 Department of Clinical Psychological Science, Faculty of Psychology and Neuroscience, Maastricht University, Maastricht, The Netherlands; 8 Department of Psychology, Institute of Life Sciences, Humboldt-Universität zu Berlin, 12489 Berlin, Germany; 9 Institute of Biometry and Clinical Epidemiology, Charité – Universitätsmedizin Berlin, 10117 Berlin, Germany; 10 neomento GmbH, 10179 Berlin, Germany; 11 Department of Neurology and Experimental Neurology, Charité – Universitätsmedizin Berlin, Campus Virchow Klinikum, Berlin, Germany; 12 Salus Clinic Lindow, 16835 Lindow, Germany; 13 TH Aschaffenburg - University of Applied Sciences, 63743 Aschaffenburg, Germany; 14 Department of Psychology, Institute for Mental Health and Behavioral Medicine, HMU Health and Medical University, 14471 Potsdam, Germany

**Keywords:** Craving, Cue reactivity, Alcohol dependence, Virtual reality, Psychophysiological parameters, Cue exposure

## Abstract

**Background:**

Craving is a diagnostic criterion and predictive factor in alcohol use disorder (AUD). In Virtual Reality Cue Exposure (VR-CE), it is intentionally induced as a basis for Virtual Reality Cue Exposure Therapy. As the underlying subjective and physiological factors and predictors of craving are still incompletely understood, we aim to identify factors associated with craving responses during VR-CE in patients with AUD.

**Methods:**

Craving responses of 61 patients with AUD during VR-CE were measured using subjective ratings (visual analogue scale, VAS) and physiological parameters, including electrodermal activity (EDA, non-specific skin conductance responses (NS.SCR) and skin conductance level (SCL)), heart rate (HR), heart rate variability (HRV), brightness corrected pupil diameter (BCPD) and respiration rate (RR). To determine factors associated with craving responses, VR-related characteristics, patients’ AUD characteristics and patients’ pre-VR craving characteristics were analyzed using linear mixed-effects models.

**Results:**

The VR-related characteristic *drink choice* (*p* = .038), several AUD-characteristics (*AUDIT score*: *p* = .019; *ADS score*: *p* < .001; *amount of alcohol consumed in the past five years*: *p* = .031) and pre-VR craving characteristics (*AUQ score*: *p* < .001; *OCDS score*: *p* < .001) significantly influenced subjective craving during VR-CE. The VR-related characteristic *type of risk scenario* (*p* = .005) influenced the HRV parameter Very Low Frequency Band (VLFB). Several AUD-characteristics (*AUDIT score*: *p* = .007; *ADS score*, *p* = .003; *days since last alcohol consumption*: *p* = .018) and the pre-VR *OCDS score* (*p* = .015) were associated with changes in RR.

**Conclusions:**

Clinical characteristics, particularly AUD severity and pre-VR craving were more consistently associated with craving responses than VR-specific factors. The limited overlap between subjective and physiological measures highlights the complexity of the concept craving and underlines the importance of using multidimensional assessments. This study contributes to a better understanding of predictors on craving and may inform the further development of optimized VR-based interventions for AUD.

**Trial registration:**

This study followed a pre-published study protocol and was preregistered on ClinicalTrials.gov (NCT05861843, Registration Date: 2023-04-24).

**Supplementary Information:**

The online version contains supplementary material available at https://doi.org/10.1186/s12888-026-08544-6.

## Background

Craving is a phenomenon with various definitions, often described as a subjective desire that motivates the consumption of a substance [1]. While alcohol craving is an extensively studied predictor for relapses [2, 3], its causes and modifiers remain incompletely understood due to the multidimensional nature of craving. In a systematic review on craving models, van Lier et al. identified several empirically supported models from cognitive, psychological, and motivational domains [4]. In cognitive behavioral therapy, craving is induced intentionally in cue exposure therapies (CET) relying on the theoretical framework of conditioning, extinction learning, and cognitive restructuring [5, 6]. The successful induction of craving is therefore important for enabling a realistic simulation of the risk situations patients face outside the hospital. Given the complexity of craving in AUD, it is beneficial to examine it in a laboratory setting. This is possible through VR that enables standardized procedures and high ecological validity [7, 8].

Virtual Reality (VR) has gained significance in the reenactment of anxiety-inducing or craving-inducing situations, forming the basis of exposure therapies for anxiety disorders, post-traumatic stress disorders, and, more recently, also substance use disorders [9]. The advantages of VR are, e.g., the accessibility and practicability in clinical routines where time, staff, and resources for in vivo exposures, especially for substance use disorders, are limited [10]. Studies by our group and others have shown that craving can be successfully induced through VR exposure to alcohol-associated risk scenarios [11, 12]. However, less is known about which factors determine the magnitude of craving responses during VR exposure. Additionally, identifying influential factors in craving is important, firstly, to develop more effective and personalized CET and, secondly, to define patient groups predominantly susceptible to these VR-based approaches.

A few studies have already examined potential influencing factors of craving in VR scenarios: Regarding suitable patient groups, Hernández-Serrano et al. concluded that especially patients with intensive craving and patients with use of illicit drugs before undergoing VR-CET benefited from this form of treatment [13]. With regard to these clinical factors, other studies that did not include VR interventions found complex and ambiguous associations between the duration of abstinence and craving: While some studies reported a negative association between duration of abstinence and craving [14] others described an “incubation of craving” during abstinence [15, 16]. Ghiță et al. investigated self-reported triggers of alcohol craving in VR scenarios. Situations associated with higher cravings were, e.g., being in a bar, pub, restaurant, at a party, or at home [17]. For the type of alcohol in VR, beer, wine, and whiskey were reported as the most effective craving triggers [17]. However, this data is based on questionnaires asking patients to imagine scenarios and rate their craving. Research on determinants of craving based on continuous, multimodal measurements during VR exposure remains limited. As craving is a complex construct with many facets, we not only include different subjective measures but also various psychophysiological measures, such as electrodermal activity (EDA), heart rate (variability), brightness corrected pupil diameter (BCPD), and respiration rate (RR), collected during VR exposure. In a previous analysis [18] we could show that exposure to alcohol-associated scenarios in VR lead to significant changes in not only subjective but also psychophysiological parameters, specifically parameters of EDA, BCPD and RR.

The primary objective of this study was to identify factors associated with craving responses during Virtual Reality cue exposure (VR-CE) in patients with alcohol use disorder (AUD). We hypothesized that characteristics of patients’ AUD (including *AUD screening score (AUDIT)*, *AUD severity score (ADS)*, *amount of alcohol consumed in the past five years* (pure alcohol, in kilogram) and *days since last alcohol consumption*) and pre-VR craving characteristics (including *Alcohol Urge Questionnaire (AUQ)*,* Obsessive Compulsive Drinking Scale (OCDS)*,* Craving Automated Scale for Alcohol (CAS-A) scores*) significantly influence the development of craving in VR. In addition, exploratory analyses examined the influence of VR-related characteristics (including *order of the exposure*,* type of risk scenario*,* drink choice*).

## Methods

### Study design

This prospective, exploratory, single-arm clinical study aimed to identify factors associated with craving which was assessed using both subjective and psychophysiological measures of craving during VR-CE in patients with AUD. Data on the effects of VR-CE on craving induction in this sample was previously published in Lütt et al. 2026 [18]. The study adhered to the study protocol [19] and was preregistered on ClinicalTrials.gov (NCT05861843).

### Ethical considerations

Approval for the study was granted by the Institutional Review Board of Charité – Universitätsmedizin Berlin (EA1/190/22, 23.05.2023). The study conformed to the principles outlined in the Declaration of Helsinki and its subsequent amendments.

### Participant recruitment

Participants, including inpatients and outpatients diagnosed with AUD according to ICD-10 [20] criteria, were recruited from the Charité – Universitätsmedizin Berlin and the Salus Clinic Lindow. After receiving comprehensive information about the study, potential participants underwent an eligibility screening by trained study personnel. In the study information we provided details on collection, storage and future use of study data including multimodal physiological data. Written informed consent was obtained from all participants, who were compensated with 30€ after study participation.

### Eligibility

Inclusion and exclusion criteria are defined in the study protocol [19] and can be found in the supplements.

### Clinical data and questionnaires

General baseline data included age, gender, demographic data, medical history, medication, and comorbidities assessed with the Charlson Comorbidity Index (CCI [21]) and the patient’s medical history. Clinical assessments followed the previously published study protocol [19]:

AUD severity was assessed using the Alcohol Dependence Scale (ADS [22, 23]), Lifetime Drinking History (LDH [24]), and Alcohol Use Disorder Identification Test (AUDIT [25]). Trait craving was measured using the Obsessive Compulsive Drinking Scale (OCDS [26]) and the Craving Automated Scale for Alcohol with its five factors and two general factors “unaware” and “nonvolitional” (CAS-A [27]). State craving was measured using the Alcohol Urge Questionnaire (AUQ, [28]).

### VR exposure

A detailed description of the VR scenarios is provided in the published study protocol [19]. A VR-head-mounted display (HTC VIVE Pro Eye) and a desktop PC based on SCHENKER XR Station with Intel Core i5-12,500 were used as VR hardware. During the VR-CE, the average ambient room temperature was 23.89 ± 2.23 (19–28.9) °C. Participants chose their preferred drink (schnaps, red wine, white wine, beer, or vodka) and one of two bars: a wine bar or a corner pub. The scenarios were then tailored to individual preferences accordingly. The session started with a short VR acclimation period (1–5 min.) in a clean, white waiting room, to help patients become familiar with the VR setup and to reduce arousal. After this acclimation, participants started with the first neutral baseline scenario consisting of a black room with a grid for orientation (5 min.) and then entered the first of two risk scenarios (5 min.), presented in pseudorandomized order. A break of 45 min. between the two sets of exposure (baseline and risk scenario) allowed craving to decrease. Then, participants resumed from a baseline scenario before entering the second risk scenario.

At the end of the session, participants were asked for residual craving and potential increased relapse risk. In the event of severe subjective craving with risk of relapse or patient endangerment, appropriate intervention would have been provided by trained study physicians and study psychologists, with additional support from colleagues from the addiction medicine ward or experienced emergency department staff if required. At no point during the study was such an intervention necessary.

### Psychophysiological parameters

The Bionomadix Wireless respiration (RSP), electrocardiogram (ECG), photo plethysmogram (PPG), and EDA wearable amplifiers, paired with a transducer connected to the Biopac MP160 data acquisition system (Biopac Systems, Santa Barbara, CA, USA), were employed to collect psychophysiological data. This system was connected to a computer running the data acquisition software AcqKnowledge 5.0. The sampling rate was set to 1000 Hz. Measurement and processing of physiological data is described in detail in Lütt et al. 2026 [18].

### Heart rate and heart rate variability

ECG signals were recorded using a standard 3-lead configuration and preprocessed using automated and manual artifact correction procedures [18].

Once the ECG signal was cleaned and all R peaks were identified, parameters were extracted from the cleaned signal over the full 5 min. duration of each VR scenario using the AcqKnowledge HRV Analysis Suite. Time-domain measures included the standard deviation of successive R-R interval differences (SDSD, ms), the root mean square of successive R-R differences (RMSSD, ms), and the percentage of R-R intervals differing by more than 50 ms (pNN50, %). Frequency-domain measures encompassed the power (ms²) within the Very Low Frequency Band (VLFB: 0.0033–0.04 Hz), Low Frequency Band (LFB: 0.04–0.15 Hz), High Frequency Band (HFB: 0.15–0.40 Hz), and Very High Frequency Band (VHFB: 0.40–3.00 Hz). Additionally, total power (ms²) and the ratios LF/(LF + HF), HF/(LF + HF), and LF/HF were calculated. Non-linear domain parameters included the Poincaré plot standard deviation perpendicular to the line of identity (SD1), the Poincaré plot standard deviation along the line of identity (SD2), and the SD1/SD2 ratio.

Cleaned R-R intervals were further processed in Python (version 3.13.0) using standard scientific libraries (pandas, NumPy, regex [29–31]), enabling calculation of the standard deviation of R-R intervals (SDRR, ms) as well as heart rate (HR, bpm). Heart rate was calculated for each one-minute segment of the VR exposure period.

### Brightness-corrected pupil diameter

Pupil diameter and VR scene brightness were monitored using the integrated eye-tracking system within the VIVE Pro Eye Headset, which records binocular data at 120 Hz with a 110° trackable field of view. Data was processed using the Soma Reality algorithm (Soma Reality GmbH, Austria, algorithm version 1.0, key parameter settings: Pupil Dilation Confidence Threshold: 0.7, Pupillary Light Reflex (PLR) Recalibration Interval: 5000ms, PLR Calibration Weight Threshold: 0.003, PLR Minimum Elements per Bin: 200), enabling the separation of illumination-induced changes from cognitively driven variations in pupil diameter, as demonstrated by Gollan et al. [32] and based on methods outlined by Beatty et al. [33]. Brightness-corrected pupil diameter (BCPD, mm) was derived from this process and pre-processed using Jupyter Notebook Version 7.0.8 [34] in the Anaconda Python distribution Version 24.11.3 [35] with the packages pandas and NumPy [29–31].

### Respiration rate

Respiration rate was measured using a transducer secured to the participant’s chest or abdomen with an adjustable nylon strap, depending on their primary breathing pattern. The respiratory signal was filtered with a Bandpass Finite Impulse Response (FIR) filter (0.05–1 Hz) to isolate relevant frequencies. From this, breaths per minute (BPM) were calculated and included in further analyses.

### Electrodermal activity

The EDA signal was obtained using disposable Ag/AgCl dry electrodes prepared with isotonic paste. Electrode placement was on the non-dominant hand on the volar surface of the medial phalanges of the index and middle fingers. The EDA signal was deconvoluted using continuous decomposition analysis from the Ledalab toolbox74 [36] in Matlab (MathWorks) Version 24.2.0.2712019 [37] into the slow-varying skin conductance level (SCL) and the frequency of non-specific skin conductance responses (NS.SCR), defined as responses exceeding a threshold of 0.03 µS (microsiemens). Participants who did not produce peaks above this threshold (“non-responders” [38, 39]) were excluded from the NS.SCR frequency analysis.

### Influencing factors of craving during VR-CE

Different categories of influencing factors of subjective and psychophysiological craving parameters during VR-CE were analyzed: The VR characteristics, specific to the technical characteristics, including the *order of the exposure*, e.g. the first and second baseline and risk scenario, the *type of risk scenario* (living room, wine bar, corner pub) with participant’s choice of the preferred bar scenario as well as participant’s *drink choice* (beer, white wine, red wine, schnaps, vodka). The second category contained participants’ AUD characteristics, including information on their *AUD screening score (AUDIT)*, their *AUD severity score (ADS)*, the *amount of alcohol consumed in the past five years* (pure alcohol, in kilogram) and *days since last alcohol consumption*. The third class of predictors included participants’ pre-VR craving characteristics, obtained by the scores of the *AUQ*,* OCDS*, and *CAS-A* questionnaires.

### Statistical methods

Statistical analyses were conducted in RStudio (Version 2024.4.0.735, Chocolate Cosmos) using R Version 4.3.2 (Eye Hole). Descriptive statistics are reported as means ± standard deviation and ranges for continuous variables, and as frequency and percentage for categorical variables.

To examine factors influencing craving during VR-CE, linear mixed-effects models (LMEs) were applied separately for subjective and psychophysiological outcome measures, with post hoc tests performed using Satterthwaite approximation to obtain t-statistics and p-values [40]. LMEs were chosen to account for the repeated-measures structure of the data and to allow inclusion of incomplete observations. Subjective craving was assessed using visual analogue scale (VAS) ratings at predefined time points (minutes 0:30, 2:30, 4:30) during baseline and risk scenario (baseline: B1, B3, B5; risk scenario: R1, R3, R5). Data on psychophysiological parameters (EDA, HR, RR and BCPD) were divided into five equal segments for each 5-minute exposure period and analyzed across these segmented time intervals within each VR condition (baseline: B1, B2, B3, B4, B5; risk scenario: R1, R2, R3, R4, R5). HRV parameters were calculated over the full duration of each scenario (baseline: B; risk scenario: R).

Assessment phase (baseline vs. risk scenario) was included as a categorical within-subject factor in all models. We further included (1) VR-related characteristics (2), AUD-related clinical variables and (3) pre-VR craving measures as fixed effects, and a random intercept to account for interindividual variability. We first explored the overall effects of predictors on subjective and psychophysiological craving parameters. Second, fixed interaction effects were added to the LMEs, analyzing each predictor separately, including a two-way interaction term between assessment phase and the respective predictor, while controlling for all remaining predictors and covariates with potential impact on physiological parameters according to guidelines and prior studies (EDA: age, ambient temperature, gender, medication [39]; HR, HRV [41] and RR [42–46]: age, ambient temperature, gender, medication, smoking status, BMI and health status (CCI-score); BCPD: medication and age [47–52]. In case of significant interaction effects, we compared the full model (including the interaction term) to a reduced model (excluding the interaction term) using the R package MuMIn [53]. This allowed for the calculation of an effect size of the interaction in terms of additional variance explained (ΔR²). We further conducted post hoc analysis of significant interactions to determine their direction. Whereas main effects show impact of predictors on craving regardless of the assessment phase (not only risk situation but also baseline), interaction effects demonstrate the influence of predictors on the change of craving from baseline to risk scenario. The direction of this association is then analyzed in the last step (post hoc): We analyzed interaction contrasts using estimated marginal means (emmeans [54]), for categorical predictors and conducted simple slope analyses at low (–1 SD), mean, and high (+ 1 SD) levels using the interactions package [55] for continuous predictors. Continuous predictors and covariates were centered within clusters and standardized as z-values. The covariate temperature was missing for one case and was hence interpolated.

Each exposure to baseline and risk scenario was obtained in one data frame, resulting in two exposures and thus two data frames per participant (baseline 1 + risk scenario 1, baseline 2 + risk scenario 2. Due to technical problems and some missing values, the following number of subjects had partial missing data in the final analysis: 1 in subjective craving, 2 in BCPD as well as 1 in HRV, HR, RR and EDA (SCL and NS.SCR). LMEs can naturally deal with longitudinal missingness which is why these subjects were not dropped from the analysis entirely but all present data could be used. NS.SCR frequency was not analyzed for two non-responders (with two data frames each). Furthermore, data were completely missing for one participant in the BCPD data, while another participant lacked all physiological data (BCPD, HR, HRV, RR, and EDA measures, including SCL and NS.SCR).

## Results

### Descriptive analysis

Relevant baseline demographic and medical characteristics for the analysis of predictors are presented in the suppl. Table 1. We used a dataset of 61 participants (49 male, 12 females) from a previous study [18]. The average age of the subjects was 48.16 ± 9.82 years (31–65).

All participants were exposed to the living room scenario and could select between two bar environments. The corner pub was chosen by 60.7% (*n* = 37), while 39.3% (*n* = 24) preferred to enter the wine bar. Additionally, participants were given the option to choose the type of drink presented in the VR-CE, 32.8% selected beer (*n* = 20), 23% chose vodka (*n* = 14), 19.7% preferred red wine (*n* = 12), 16.4% chose white wine (*n* = 10), and 8.2% selected schnaps (*n* = 5). A Kruskal-Wallis test revealed no significant differences in dependence severity across beverage categories, χ^2^ (4) = 4.71, df = 4, p-value = 0.319.

### Effects of predictors on subjective craving during VR-CE

LME models revealed significant main and interaction effects of the predictors on the subjective craving (ps < 0.05). Significant main effects were observed for the VR characteristic *order of the VR-CE* (e.g., first bar, then living room), the AUD characteristic *days since last alcohol consumption* and the pre-VR craving characteristic *AUQ score* (ps < 0.05, supp. Table 3). For an overview of the significant interaction effects, which are then explained in detail in the following paragraphs, see Table 1. Post hoc simple slopes analysis on significant interaction effects revealed an increase across all levels of the respective predictor, hence we focus on the steepest slope to answer the question if the highest increase in craving is associated with higher or lower values of the predictor. The LME model including only the main effects explained R² = 0.608 of the variance in subjective craving. The inclusion of interaction terms resulted in small increases in explained variance in subjective craving.

**Table 1:** Overview of significant interaction effects and their influence on subjective craving

Predictor class	Predictor	Interaction	Influence on subjective craving
VR characteristics	*Drink choice*	×R3 & R5	Steepest increase in patients choosing schnaps compared to all other drinks
AUD characteristics	*AUDIT score*	×R5	Increase across all levels of the respective predictor; Steepest increase in high values (+ 1SD) of the respective predictor
	*ADS score*	×R3 & R5	
	*amount of alcohol consumed in the past five years* (pure alcohol, in kilogram)	×R3 & R5	
Pre-VR craving characteristics	*AUQ score*	×R3 & R5	
	*OCDS score*	×R1, R3 & R5	

Annotation. Only significant interaction effects and simple slopes analysis are displayed

### VR characteristics

**Fig. 1:**
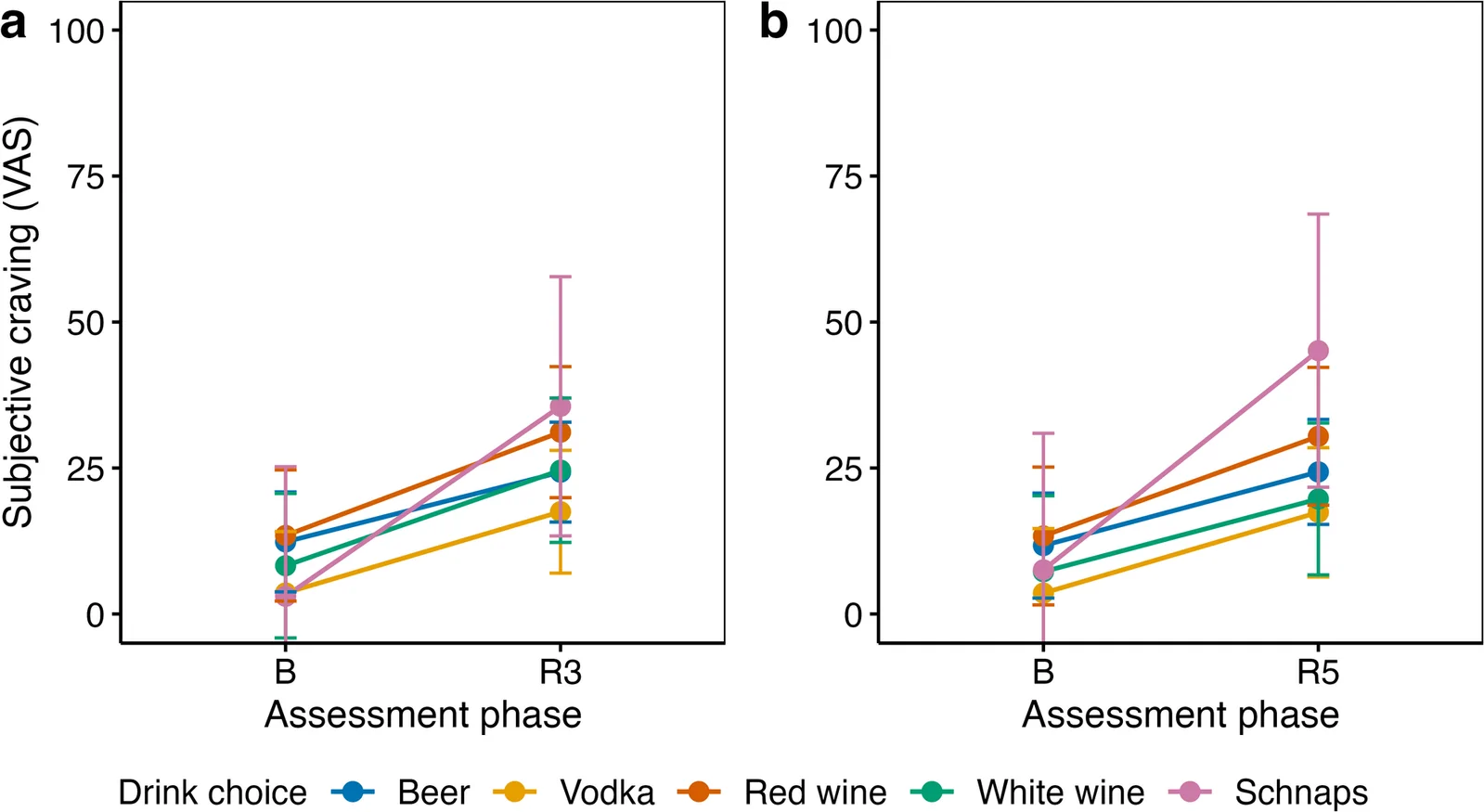
Linear mixed-effect models displaying interaction effects of *drink choice* × VR-CE assessments at R3 (**a**) and R5 (**b**) on subjective craving measured by visual analogue scale (VAS). Plots of estimated marginal means and 95% confidence intervals of the means obtained from linear mixed-effect models displaying significant interaction effects by VR-CE assessments and *drink choice* on subjective craving

The LME models including interaction terms revealed a significant assessment phase × *drink choice* interaction, F(24, 752.99) = 1.58, *p* = .038, with the full model including the interaction yielding a ΔR² of 0.018. During risk assessment phase R3 and R5, participants selecting schnaps exhibited a significantly greater increase in subjective craving from baseline to risk assessment relative to all other participants who chose beer, white wine, red wine, or vodka (Fig. 1, supp. Table 4). Due to the small subgroup of participants selecting schnaps (*n* = 5), this finding should be treated as exploratory. No significant interaction effects emerged for *order of the VR-CE* and *type of risk scenario* (ps > 0.05, supp. Table 3).

### AUD characteristics

A significant assessment phase × AUD characteristics interaction emerged for the *AUDIT score*, F(6, 770.99) = 2.55, *p* = .019, with ΔR² = 0.007, with significant post hoc interaction contrasts from baseline to risk assessment phase R5 showing the influence of the predictor *AUDIT score* on the increase of craving from baseline to risk scenario. Furthermore, significant interactions were observed for the *ADS score*, F(6, 770.99) = 3.93, *p* < .001, with ΔR² = 0.011, and *amount of alcohol consumed in the past five years* (pure alcohol, in kilogram), F(6, 770.99) = 2.33, *p* = .031, with ΔR² = 0.007 and significant post hoc interaction contrasts from baseline to risk assessment phase R3 and R5 for both predictors. Simple slopes analysis showed increased subjective craving from baseline to risk scenario across all levels (low: -1 SD, mean, and high: +1 SD) of *AUDIT scores* (suppl. Figure 1), *ADS scores* (suppl. Figure 2) and *amount of alcohol consumed in the past five years* (pure alcohol, in kilogram, suppl. Figure 3), with the steepest increase at high (+ 1 SD) levels. This shows that the highest values of the aforementioned predictors had the greatest influence on the increase in cravings. No significant interaction effects were revealed for *days since last alcohol consumption* (ps > 0.05, supp. Table 3).

### Pre-VR craving characteristics

**Fig. 2:**
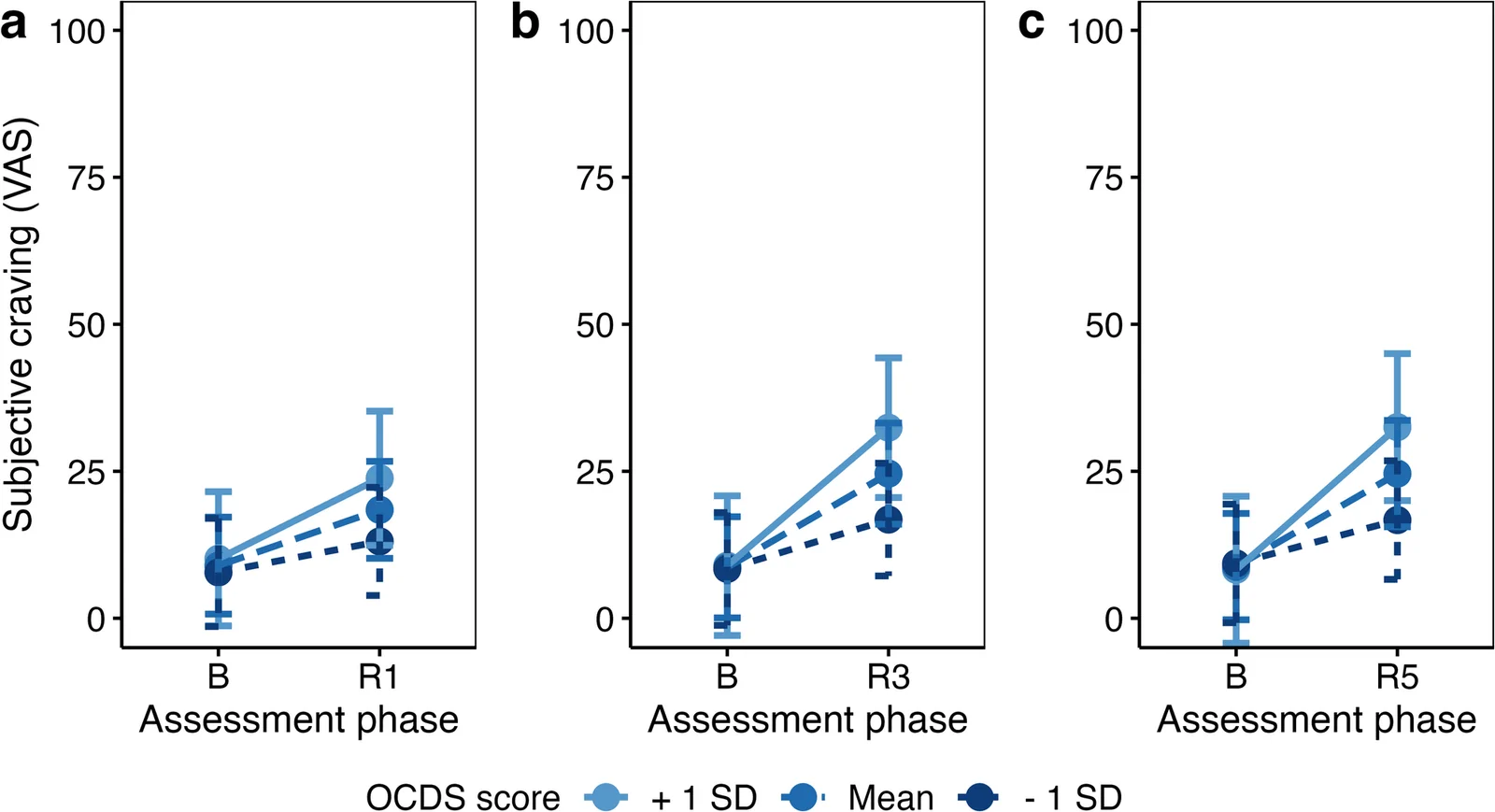
Linear mixed-effect models displaying interaction effects of *OCDS score* × VR-CE assessments at R1 (**a**), R3 (**b**) and R5 (**c**) on subjective craving measured by visual analogue scale (VAS). Lines represent model-estimated simple slopes at low (− 1 SD), mean, and high (+ 1 SD) levels of *OCDS score*. Error bars indicate 95% confidence intervals

The LME models including interaction terms revealed significant interaction effects of assessment phase × pre-VR craving characteristics (ps < 0.05), except for the two *CAS-A factors* (ps > 0.05, supp. Table 3). For the predictor *AUQ score*, a significant interaction with assessment was found, F(6, 770.99) = 6.61, *p* < .001, with ΔR² = 0.018 and significant post hoc interaction contrasts from baseline to risk assessment phase R3 and R5. For the predictor *OCDS score*, a significant interaction with assessment phase emerged, F(6, 770.99) = 10.12, *p* < .001, with ΔR² = 0.027 with significant post hoc interaction contrasts across all risk scenario assessments. Simple slopes analysis again showed increased subjective craving from baseline to risk scenario across all levels of *AUQ scores* and *OCDS scores* (-1 SD, mean, + 1SD), with high (+ 1 SD) *OCDS scores* (Fig. 2) and *AUQ scores* (suppl. Figure 4) being associated with a steeper rate of increase in subjective craving (supp. Table 4).

### Effects of predictors on the physiological parameters EDA, HR, HRV, RR and BCPD during VR-CE

LME analyses revealed significant main effects on several psychophysiological craving parameters (ps < 0.05). VR-related characteristics showed significant main effects on HR, EDA (SCL & NS.SCR frequency), RR, and HRV parameters (ps < 0.05). Additional main effects of AUD characteristics were observed on BCPD, NS.SCR frequency, HR and HRV parameters (ps < 0.05), except for *amount of alcohol consumed in the past five years* (pure alcohol, in kilogram, ps > 0.05). Pre-VR craving characteristics showed significant main effects on NS.SCR frequency, HR and HRV parameters (ps < 0.05), with the exception of *CAS-A g-factor 2* (“non-volitional”, ps > 0.05; supp. Tables 6 and 7). In contrast, significant assessment phase × predictor interaction effects were limited and observed only for RR and the HRV parameter VLFB (ps < 0.05; supp. Tables 6 and 7). The LME model including only the main effects explained R²=0.612 of the variance in RR and R²=0.322 of the variance in VLFB with the inclusion of interaction terms resulting in additional small increases in explained variance. For an overview, see Table 2.

**Table 2:** Overview of significant interaction effects and their influence on physiological craving parameters

Physiological Parameter	Predictor class	Predictor	Interaction	Influence on physiological craving parameters
VLFB (HRV parameter)	VR characteristics	*Type of risk scenario*	×R	Decrease in the pub condition compared to the living room and wine bar
RR	AUD characteristics	*AUDIT score*	×R4	Increase across low (-1SD) and mean values of the respective predictor; Steepest increase in low values (-1SD) of the respective predictor
		*ADS score*	×R3, R4, R5	
		*Days since last alcohol consumption*	×R1	Increase across all levels of the respective predictor; Steepest increase in high values (+1SD) of the respective predictor
	Pre-VR craving characteristics	*OCDS score*	×R1	Increase across all levels of the respective predictor; Steepest increase in low values (-1SD) of the respective predictor

Annotation: only significant interaction effects and significant simple slopes analysis are displayed

### VR characteristics

**Fig. 3:**
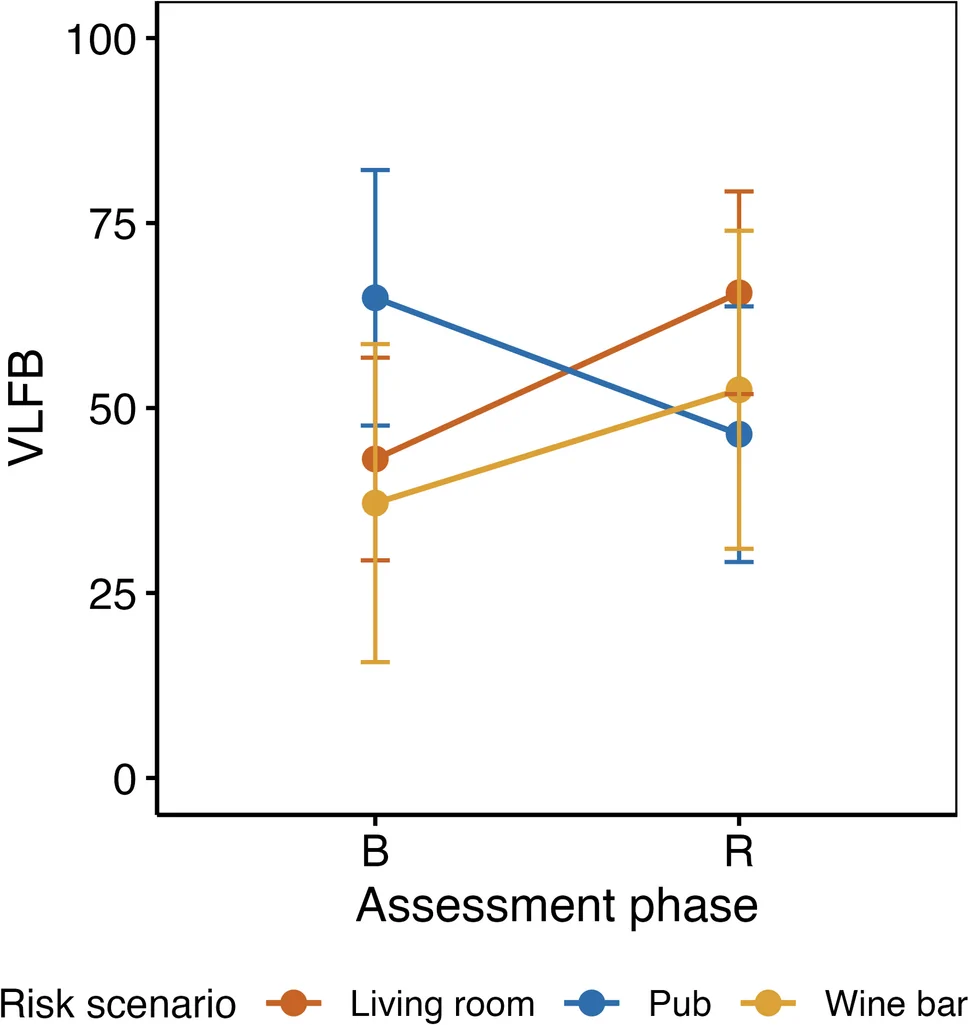
Linear mixed-effect models displaying interaction effects of *type of risk scenario* × VR-CE assessments at risk situation on the HRV parameter Very Low Frequency Band (VLFB). Plots of estimated marginal means and 95% confidence intervals of the means obtained from linear mixed-effect models displaying significant interaction effects by VR-CE assessments and *type of risk scenario* on VLFB

Models including assessment phase × VR-characteristics interactions yielded a significant assessment phase × *type of risk scenario* interaction solely for the HRV parameter VLFB, F(2, 171.65) = 5.47, *p* = .005, with ΔR² = 0.020. Post hoc interaction contrasts revealed that patients exposed to the pub condition showed a significant decrease in VLFB from baseline to risk assessment compared to those exposed to the wine bar and living room conditions (Fig. 3, supp. Table 8). No significant interaction effects were observed for the predictor *drink choice* and *order of the VR-CE* (ps > 0.05, supp. Tables 6 and 7).

### AUD characteristics

Significant assessment phase × AUD characteristics predictor interaction effects were observed exclusively for RR (ps < 0.05): For the predictor *AUDIT score*, a significant interaction with assessment was found, F(10, 1235.02) = 2.42, *p* = .007, with ΔR² = 0.007 and significant post hoc interaction contrasts from baseline to risk assessment phase R4. Furthermore, a significant interaction with assessment was found for the predictor *ADS score*, F(10, 1235.02) = 2.65, *p* = .003, with ΔR² of 0.008 and significant post hoc interaction contrasts from baseline to risk assessment phases R3, R4 and R5. Simple slopes analysis indicated that low (-1 SD) and mean *AUDIT scores* (suppl. Figure 5) and *ADS scores* (suppl. Figure 6) were associated with an increase in RR from baseline to risk scenario, with the steepest increase at low (-1 SD) values. For the predictor *days since last alcohol consumption*, a significant interaction with assessment was found, F(10, 1235.02) = 2.17, *p* = .018, with ΔR² of 0.006 and significant post hoc interaction contrasts from baseline to risk assessment phase R1. Simple slopes analysis indicated an increase of RR from baseline to risk scenario across all levels of *days since last alcohol consumption* (-1 SD, mean, + 1 SD). More (+ 1 SD) *days since last alcohol consumption* were associated with a steeper rate of increase (suppl. Figure 7, supp. Table 9). No significant interaction effects were observed for the predictor *amount of alcohol consumed in the past five years* (pure alcohol, in kilogram, ps > 0.05, supp. Tables 6 and 7).

### Pre-VR craving characteristics

Significant assessment phase × pre-VR craving characteristics interaction effects again only emerged for RR (*p* = .015): Specifically, only the assessment phase × *OCDS score* interaction was significant, F(10, 1235.02) = 2.21, *p* = .015, with a ΔR² of 0.007. Post hoc interaction contrasts were significant from baseline to the risk assessment phase R1. Simple slopes analysis indicated an increase in RR from baseline to risk scenario across all levels of *OCDS scores*, with lower (-1 SD) *OCDS scores* associated with a steeper rate of RR increase (suppl. Figure 8, supp. Table 9). No significant interaction effects were observed for the predictors *AUQ score*,* CAS-A score* g-factor 1 (“unaware”), and 2 (“non-volitional”) (ps > 0.05, supp. Tables 6 and 7).

## Discussion

### Principal findings

#### Subjective craving

This study identified several factors associated with subjective craving during VR-CE in patients with AUD.

Regarding VR characteristics, the participant’s *drink choice* influenced the change in subjective craving from baseline to the risk scenario (interaction effect): those who chose schnaps showed a steeper increase in subjective craving than those who chose beer, white wine, red wine, or vodka.

AUD-related variables, including *amount of alcohol consumed in the past five years*, *AUDIT scores*, and *ADS scores*, were associated with increases in subjective craving from baseline to risk scenarios. Baseline craving measures (*AUQ* and *OCDS)* were also associated with changes in subjective craving, with *OCDS scores* showing the strongest contribution.

Notably, interaction effects were primarily observed at later stages of the risk scenarios (R3 and R5), suggesting that craving responses develop over time rather than occurring immediately upon exposure. Within the interaction effects, increases in subjective craving were observed across all levels of the respective predictors, with the steepest increases occurring at higher values of the predictors.

#### Physiological craving parameter

In contrast to subjective measures, predictors influencing changes in physiological parameters were limited and observed for respiration rate (RR) and VLFB. For the VR characteristic, the *type of risk scenario* influenced the change in the HRV parameter VLFB from baseline to the risk scenario (interaction effect), with a decrease in the pub condition compared to the living room and wine bar, potentially reflecting a decreased physiological response to alcohol cues in this condition [56].

Clinical characteristics related to AUD also influenced physiological craving parameters during VR-CE: The duration of abstinence further influenced the change of participants’ RR from baseline to risk scenario, with more *days since last alcohol consumption* being associated with a steeper rate of increase of RR. In addition, participants’ *AUDIT score* and *ADS score* influenced the change of participants’ RR from baseline to risk scenario: low values resulted in the steepest increase in RR. Baseline craving measures prior to VR-CE, specifically, participants’ *OCDS score*, further influenced the change of participants’ RR from baseline to risk scenario, with a steeper rate of increase of RR at lower *OCDS scores*.

#### Similarities of influencing factors of both subjective and physiological craving parameters

Only a limited overlap was observed between predictors influencing subjective and physiological craving measures. This dissociation supports the notion that craving is a multidimensional construct, with subjective and physiological components reflecting partially distinct processes [1, 57]. These were observed mainly for RR, whereas other predictors showed a significant influence on either the subjective or psychophysiological side. Only the AUD characteristics *AUDIT scores* and *ADS scores*, as well as the pre-VR craving characteristic *OCDS score*, influenced changes in both subjective and physiological craving parameters from baseline to risk scenario during VR-CE (interaction effect).

### Comparison to prior literature

One strength of this study is its multimodal measurement of craving parameters in patients during actual exposure to the VR paradigms. The above-mentioned study by Ghiță et al. [17] focused on self-reports on hypothetical craving without patients being exposed to the named situations.

Hernández-Serrano et al. [13] compared craving levels pre and post VR-CET and treatment as usual (TAU) against patients receiving TAU and found that being in the group receiving VR-CET + TAU and illicit drug use prior to therapy increased the odds of having an improvement (decrease) in subjective craving over the course of therapy. However, the design is not comparable to our study, which focused on craving in one session, measured at different time points, and on a physiological level, in addition to subjective reports.

In a later study in 2021, Hernández-Serrano et al. [58] analyzed determinants of subjective craving and perceived realism in one session of VR-CE, again excluding physiological parameters. Here, AUD severity and perceived realism of virtual drinks were found to be predictors of subjective craving. A mediational model showed that perceived realism had a mediational effect on the relationship between AUD severity and alcohol craving.

Interestingly, the significant interaction effect between AUD severity and craving could be confirmed in our study, not only on a subjective but also physiological level. While higher disease severity was also associated with higher subjective craving in our study, the results in relation to RR surprisingly showed an inverse correlation: Lower ADS scores, lower baseline craving scores and a longer abstinence were associated with higher increase in RR. It should be noted, however, that this was limited to segments of R and not throughout the risk situations (R1 - R5). Additionally, as depicted in e.g. suppl. Figure 6, this could be in part explained by higher baseline RR in the group with the highest scores in ADS as one example. This discrepancy between subjective craving parameters and RR as a physiological marker, as well as the other not significant physiological predictors, may also be associated with a reduced interoceptive accuracy and recall difficulties of AUD patients [59]. However, this does not explain the observation that longer abstinence duration was associated with a steeper increase in RR. Furthermore, RR may be influenced by other emotional or somatic covariates (anxiety, body temperature) although our analyses already adjusted for a broad range of potential covariates as stated above.

An early study by Reid et al. [60] on subjective and physiological craving after cue exposure to non-VR alcohol cues showed a correlation of subjective craving with baseline measures of recent alcohol craving and severity of AUD, which could be confirmed by our study. Number of days since last alcohol use did not correlate significantly with craving in their and our study. While RR was not measured, they could show a significant correlation between salivation, a parameter not collected by us, drinking years (positive correlation) and self-efficacy (negative correlation) [60]. Subjective and physiological craving parameters were not significantly correlated, in contrast to our recently published study [18] which showed weak but significant correlations between subjective craving and EDA measures.

An extension compared to their study and many previous studies is the inclusion of VR characteristics as possible influencing factors on subjective and on physiological parameters. However, our results do not allow for clear conclusions based on the influence of both (subjective and physiological) parameters that could serve for the development of future VR scenarios.

### Limitations

Several limitations should be considered. The single-center single-arm design of the present study with only one session precludes statements about the change of influential factors over the course of a VR-CET. However, this would be a next step in future studies which should also include healthy control groups. Prior VR experience was not assessed in participants and could have influenced physiological responses. We tried to avoid this by employing a VR acclimation room before entering the study paradigm. The exploratory nature of the analyses with multiple comparisons without correction and the large number of predictors increase the risk of type I error. Additionally, effect sizes were generally small, limiting the clinical interpretability of individual predictors. Subgroup analyses (e.g., drink choice) were based on small sample sizes. Finally, psychophysiological measures are inherently variable and may be influenced by factors not fully captured in the present study.

## Conclusions

In this study we analyzed based on a complex multimodal experimental design incorporating various subjective and psychophysiological parameters, the different levels of influencing factors on craving, covering both VR- and participant characteristics. Patient-related characteristics, particularly baseline craving and AUD severity, were more consistently associated with craving responses during VR-CE than VR-specific factors. The limited overlap between subjective and physiological measures highlights the complexity of craving and challenges the use of single-modality assessments. Although effect sizes were small, these findings contribute to a better understanding of craving mechanisms and provide a basis for future research on more individualized VR-based interventions in AUD.

## Supplementary Information

Below is the link to the electronic supplementary material.

Supplementary Material 1

## Data Availability

Due to the sensitive nature of the clinical data and the restrictions imposed by the Charité - Universitätsmedizin Berlin’s data protection regulations and the ethics committee approval (EA1/190/22), the individual-level data underlying this study cannot be made publicly available. Aggregated data supporting the findings of this study are available within the paper and its supplementary materials. Analysis code (R scripts for LME models) is available upon reasonable request to ensure reproducibility of the statistical analyses.

## Abbreviations

ADS: Alcohol Dependence Scale
AUQ: Alcohol Urge Questionnaire
AUD: Alcohol Use Disorder
AUDIT: Alcohol Use Disorders Identification Test
BCPD: Brightness-Corrected Pupil Diameter
BMI: Body Mass Index
CAS-A: Craving Automated Scale for Alcohol
CCI: Charlson Comorbidity Index
CET: Cue Exposure Therapy
EDA: Electrodermal Activity
FMSS: Fast Motion Sickness Scale
HR: Heart Rate
HRV: Heart Rate Variability
IPQ: Igroup Presence Questionnaire
LDH: Lifetime Drinking History
LME: Linear Mixed-Effects Model
NS.SCR: Non-Specific Skin Conductance Responses
OCDS: Obsessive Compulsive Drinking Scale
RR: Respiration Rate
SCL: Skin Conductance Level
VAS: Visual Analogue Scale
VR: Virtual Reality
VR-CE: Virtual Reality Cue Exposure
VR-CET: Virtual Reality Cue Exposure Therapy
VLFB: Very Low Frequency Band
