# Supplementary material for: Predictors of subjective and physiological craving during VR-cue exposure in alcohol use disorder: an explorative single-arm clinical study
Source: BMC Psychiatry. 2026 Aug 26;26:699. doi: 10.1186/s12888-026-08544-6 (PMC13543422; doi:10.1186/s12888-026-08544-6)
Supplement: Supplementary file 1 [file 12888_2026_8544_MOESM1_ESM.docx]

## Supplementary material

**Eligibility**

Patients were included in the study if they met the following criteria:

Inclusion criteria

- Age 18–65 years
- Diagnosis of alcohol dependence (ICD-10: F10.2)
- Completed inpatient withdrawal treatment within the past 3 months
- History of alcohol craving, confirmed via craving questionnaires
- Ability to provide written informed consent

Exclusion criteria:

- Substance dependence other than alcohol or nicotine
- Current alcohol intoxication (randomly tested via measurement of breath alcohol concentration)
- Abstinence for less than 7 days or ongoing alcohol consumption
- Severe neuropsychiatric disorder (e.g., schizophrenia spectrum disorders, bipolar affective disorder, or substantial cognitive impairment)
- Significant medical conditions affecting brain or heart function that may influence physiological study parameters
- Acute suicidality or imminent risk of endangering others
- Concurrent pharmacological treatment targeting AUD (i.e, benzodiazepines) or craving (i.e, acamprosate, disulfiram, naltrexone, nalmefene) and further medication significantly influencing heart frequency
- Inability to understand the study procedures or provide consent

**Suppl. Table 1. Baseline demographic and medical characteristics (N=61)**

| Variables | | | n (%) or  mean ± SD  (range)^a^ |
| --- | --- | --- | --- |
|  | | | |
| **Age (years)*** | | | 48.16 ± 9.82 (31 - 65) |
| **Gender*** | | |  |
|  | Male* | | 49 (80.3) |
|  | Female* | | 12 (19.7) |
| **BMI*** | | | 26.92 ± 5.84 (18.8 - 48.8) |
| **AUD characteristics** | | |  |
|  | Days since last alcohol consumption* | | 20.64 ± 22.42 (7 - 109) |
|  | Alcohol consumption in the past 5 years (kilogram)* | | 342.70 ± 301.64 (112.26 - 1421.12) |
|  | AUD identification score (AUDIT) | | 27.31 ± 7.14 (11 - 40) |
|  | AUD severity score (ADS) | | 20.8 ± 8.11 (5 - 40) |
| **Comorbidities*** | | |  |
|  | Yes | | 38 (62.3) |
|  |  | CCI-score | 0.93 ± 1.08 (0 - 4) |
|  | No | | 23 (37.7) |
| **Medication** | | |  |
|  | Yes* | | 39 (63.9) |
|  |  | Medication Class^b^ |  |
|  |  | SNDRI | 1 (1.35) |
|  |  | SSRI | 9 (12.16) |
|  |  | SSNRI | 4 (5.41) |
|  |  | NaSSA | 4 (5.41) |
|  |  | TCA | 0 (0.00) |
|  |  | Antipsychotics^c^ | 5 (6.76) |
|  |  | Anxiolytics | 0 (0.00) |
|  |  | Antihypertensives | 20 (27.03) |
|  |  | Anticonvulsants | 2 (2.7) |
|  |  | Antidiabetics | 5 (6.76) |
|  |  | Analgesics | 2 (2.7) |
|  |  | Stimulants | 1 (1.35) |
|  | No* | | 22 (36.1) |
| **Smoking*** | | |  |
|  | Yes | | 45 (73.8) |
|  | No | | 16 (26.2) |
| **Pre-VR craving characteristics** | | |  |
|  | AUQ score | | 14.66 ± 7.78 (8 - 38) |
|  | OCDS score | | 25.8 ± 8.68 (12 - 45) |
|  | CAS-A g-factor 1 (“unaware”) | | 21.97 ± 13.24 (0 - 57) |
|  | CAS-A g-factor 2 (“non-volitional”) | | 38.82 ± 15.57 (2 - 74) |
| ^a^Categorical variables are presented as n (%; rounded to whole numbers), and continuous variables as mean ± SD (range)  ^b^free-text response item → percentage of n=39  ^c^n=3 Quetiapin, n=1 Pipamperon, n=1 Melperon  *Lütt et al., 2026 (1)  CCI: Charlson Comorbidity Index, SNDRI: serotonin–norepinephrine–dopamine reuptake inhibitor, SSRI: selective serotonin reuptake inhibitors, SSNRI: selective serotonin reuptake inhibitors, NaSSA: noradrenergic and specific serotonergic antidepressant, TCA: tricyclic antidepressant | | | |

**Supplementary table 2. Linear mixed effects models of main and interaction effects analysis for subjective craving**

**Model formula**

| **Main Effects** | | |
| --- | --- | --- |
| subjective craving ~ | Dependent variable | |
| assessment phase + | Main effects | Fixed term |
| order of the exposure + type of risk scenario + drink choice + | Main effects of VR characteristics | Fixed term |
| AUD screening score (AUDIT) + AUD severity score (ADS) + amount of alcohol consumed in the past five years + days since last alcohol consumption + | Main effects of AUD characteristics | Fixed term |
| AUQ score + OCDS score + CAS g-factor 1 („unaware“) + CAS g-factor 2 („non-volitional“) + | Main effects of pre-VR craving characteristics | Fixed term |
| (1 \| participant) |  | Random term |
| **Interaction effects**  Each predictor was analyzed separately, including a two-way interaction term between assessment and the respective predictor. | | |
| subjective craving ~ | Dependent variable | |
| assessment phase + order of the exposure + type of risk scenario + drink choice + AUD screening score (AUDIT) + AUD severity score (ADS) + amount of alcohol consumed in the past five years + days since last alcohol consumption + AUQ score + OCDS score + CAS g-factor 1 („unaware“) + CAS g-factor 2 („non-volitional“) + | Main effects | Fixed term |
| assessment phase × predictor + | Interaction effect |  |
| (1 \| participant) |  | Random term |

**Supplementary table 3. Main and interaction effect analysis for subjective craving**

**Model formula:** see supplementary table 2.

**Model Output**

| Predictor class | Predictor | Main effect analysis | |  | Interaction effect analysis | | |
| --- | --- | --- | --- | --- | --- | --- | --- |
|  |  | Regression analysis | |  | Regression analysis | | |
|  |  | Regression | p-Value |  | Regression | p-Value | ΔR² |
| VR characteristics | Type of risk scenario | F(2, 801.23) = 0.77 | .464 |  | F(12, 764.99) = 0.49 | .920 |  |
|  | Drink choice | F(4, 48.21) = 0.62 | .651 |  | F(24, 752.99) = 1.58 | .038 | .018 |
|  | Order of the exposure | F(1, 783.04) = 8.92 | .003 |  | F(6, 770.99) = 0.23 | .966 |  |
| AUD characteristics | AUD screening score (AUDIT) | F(1, 47.84) = 1.59 | .214 |  | F(6, 770.99) = 2.55 | .019 | .007 |
|  | AUD severity score (ADS) | F(1, 47.88) = 1.20 | .279 |  | F(6, 770.99) = 3.93 | <.001 | .011 |
|  | Amount of alcohol consumed in the past five years | F(1, 47.97) = 1.07 | .307 |  | F(6, 770.99) = 2.33 | .031 | .007 |
|  | Days since last alcohol consumption | F(1, 47.85) = 8.68 | .005 |  | F(6, 770.99) = 0.37 | .901 |  |
| Pre-VR craving characteristics | AUQ score | F(1, 47.91) = 10.56 | .002 |  | F(6, 770.99) = 6.61 | <.001 | .018 |
|  | OCDS score | F(1, 48.07) = 1.38 | .246 |  | F(6, 770.99) = 10.12 | <.001 | .027 |
|  | CAS-A g-factor 1 („unaware“) score | F(1, 47.87) = 0.68 | .414 |  | F(6, 770.99) = 0.17 | .985 |  |
|  | CAS-A g-factor 2 („non-volitional“) score | F(1, 47.91) = 0.11 | .739 |  | F(6, 770.99) = 0.75 | .611 |  |

**Supplementary table 4. Post hoc analysis of significant interaction effects for subjective craving during VR-CE**

**Model formula:** see supplementary table 2.

**Model Output**

| Predictor class | Predictor | Post hoc analysis of significant interaction effects | | | | | | | |
| --- | --- | --- | --- | --- | --- | --- | --- | --- | --- |
|  |  |  | Interaction contrasts | | |  |  | Simple slope analysis | |
|  |  | Inter-action | Contrasts^1^ | β (95% CI) | p-Value |  |  | β (95% CI) | p-Value |
| VR characteristics | Drink choice | ×R1 | Beer | -5.72 (-13.62, 2.19) | .311 |  |  |  |  |
|  |  |  | Vodka | -1.34 (-10.41, 7.74) | 1.000 |  |  |  |  |
|  |  |  | Red wine | 1.34 (-8, 10.67) | 1.000 |  |  |  |  |
|  |  |  | White wine | 1.62 (-8.55, 11.79) | 1.000 |  |  |  |  |
|  |  |  | Schnaps | 4.10 (-9.08, 17.27) | 1.000 |  |  |  |  |
|  |  | ×R3 | Beer | -6.53 (-14.43, 1.38) | .133 |  |  |  |  |
|  |  |  | Vodka | -4.65 (-13.73, 4.43) | .560 |  |  |  |  |
|  |  |  | Red wine | -0.77 (-10.11, 8.56) | 1.000 |  |  |  |  |
|  |  |  | White wine | -2.12 (-12.29, 8.05) | 1.000 |  |  |  |  |
|  |  |  | Schnaps | 14.07 (0.89, 27.24) | .030 |  |  |  |  |
|  |  | ×R5 | Beer | -6.14 (-14.05, 1.76) | .180 |  |  |  |  |
|  |  |  | Vodka | -4.63 (-13.71, 4.45) | .376 |  |  |  |  |
|  |  |  | Red wine | -1.69 (-11.03, 7.65) | .640 |  |  |  |  |
|  |  |  | White wine | -6.30 (-16.47, 3.86) | .329 |  |  |  |  |
|  |  |  | Schnaps | 18.77 (5.6, 31.95) | .001 |  |  |  |  |
| AUD characteristics | AUD screening score (AUDIT) | ×R5 | | 4.73 (1.32, 8.14) | .007 |  | -1 SD | 11.07 (6.24, 15.91) | <.001 |
|  |  |  |  |  |  |  | Mean | 15.82 (12.4, 19.23) | <.001 |
|  |  |  |  |  |  |  | +1 SD | 20.56 (15.73, 25.39) | <.001 |
|  | AUD severity score (ADS) | ×R3 | | 4.31 (0.92, 7.7) | .013 |  | -1 SD | 11.61 (6.8, 16.41) | <.001 |
|  |  |  |  |  |  |  | Mean | 15.93 (12.53, 19.33) | <.001 |
|  |  |  |  |  |  |  | +1 SD | 20.25 (15.45, 25.06) | <.001 |
|  |  | ×R5 | | 5.61 (2.22, 9) | .001 |  | -1 SD | 10.18 (5.38, 14.99) | <.001 |
|  |  |  |  |  |  |  | Mean | 15.82 (12.42, 19.21) | <.001 |
|  |  |  |  |  |  |  | +1 SD | 21.45 (16.64, 26.25) | <.001 |
|  | Amount of alcohol consumed in the past five years | ×R3 | | 3.84 (0.42, 7.26) | .028 |  | -1 SD | 12.09 (7.26, 16.93) | <.001 |
|  |  |  |  |  |  |  | Mean | 15.93 (12.51, 19.35) | <.001 |
|  |  |  |  |  |  |  | +1 SD | 19.77 (14.93, 24.6) | <.001 |
|  |  | ×R5 | | 4.03 (0.6, 7.45) | .021 |  | -1 SD | 11.79 (6.95, 16.63) | <.001 |
|  |  |  |  |  |  |  | Mean | 15.82 (12.4, 19.23) | <.001 |
|  |  |  |  |  |  |  | +1 SD | 19.84 (15, 24.68) | <.001 |
| Pre-VR craving characteristics | AUQ score | ×R3 | | 6.83 (3.47, 10.18) | <.001 |  | -1 SD | 9.08 (4.32, 13.84) | <.001 |
|  |  |  |  |  |  |  |  |  |  |
|  |  |  |  |  |  |  | Mean | 15.93 (12.57, 19.3) | <.001 |
|  |  |  |  |  |  |  | +1 SD | 22.78 (18.02, 27.54) | <.001 |
|  |  | ×R5 | | 6.29 (2.94, 9.65) | <.001 |  | -1 SD | 9.5 (4.74, 14.26) | <.001 |
|  |  |  |  |  |  |  | Mean | 15.82 (12.45, 19.18) | <.001 |
|  |  |  |  |  |  |  | +1 SD | 22.13 (17.37, 26.89) | <.001 |
|  | OCDS score | ×R1 | | 4.19 (0.88, 7.5) | .013 |  | -1 SD | 5.28 (0.58, 9.98) | .028 |
|  |  |  |  |  |  |  | Mean | 9.48 (6.16, 12.81) | <.001 |
|  |  |  |  |  |  |  | +1 SD | 13.69 (8.99, 18.39) | <.001 |
|  |  | ×R3 | | 7.49 (4.18, 10.8) | <.001 |  | -1 SD | 8.41 (3.71, 13.11) | <.001 |
|  |  |  |  |  |  |  | Mean | 15.93 (12.61, 19.25) | <.001 |
|  |  |  |  |  |  |  | +1 SD | 23.45 (18.76, 28.15) | <.001 |
|  |  | ×R5 | | 8.39 (5.08, 11.7) | <.001 |  | -1 SD | 7.39 (2.69, 12.09) | .002 |
|  |  |  |  |  |  |  | Mean | 15.82 (12.49, 19.14) | <.001 |
|  |  |  |  |  |  |  | +1 SD | 24.24 (19.55, 28.94) | <.001 |
| ^1^ Contrasts of the interaction effect of two categorical predictors using effect coding. | | | | | | | | | |

**Supplementary table 5. Linear mixed effects models of main and interaction effects analysis for physiological craving parameters**

**Model formula**

| **Main Effects**  Covariates with protentional influence on physiological parameters were incorporated for each physiological parameter separately according to prior research and established guidelines. | | | |
| --- | --- | --- | --- |
| Physiological craving parameter ~ | | Dependent variable | |
| assessment phase + | | Main effects | Fixed term |
| order of the exposure + type of risk scenario + drink choice + | | Main effects of VR characteristics |  |
| AUD screening score (AUDIT) + AUD severity score (ADS) + amount of alcohol consumed in the past five years + days since last alcohol consumption + | | Main effects of AUD characteristics |  |
| AUQ score + OCDS score + CAS g-factor 1 („unaware“) + CAS g-factor 2 („non-volitional“) + | | Main effects of pre-VR craving characteristics |  |
|  | age + ambient temperature + gender + medication + | Covariates with protentional influence on NS.SCR frequency & SCL |  |
|  | age + ambient temperature + gender + medication + smoking status + BMI + health status (CCI-score) + | Covariates with protentional influence on HR & RR |  |
|  | age + medication + | Covariates with protentional influence on BCPD |  |
| (1 \| participant) | |  | Random term |
| **Interaction effects**  Each predictor was analyzed separately, including a two-way interaction term between assessment and the respective predictor. Covariates with protentional influence on physiological parameters were incorporated for each physiological parameter separately according to prior research and established guidelines. | | | |
| subjective craving ~ | | Dependent variable | |
| assessment phase + order of the exposure + type of risk scenario + drink choice + AUD screening score (AUDIT) + AUD severity score (ADS) + amount of alcohol consumed in the past five years + days since last alcohol consumption + AUQ score + OCDS score + CAS g-factor 1 („unaware“) + CAS g-factor 2 („non-volitional“) + | | Main effects | Fixed term |
|  | age + ambient temperature + gender + medication + | Covariates with protentional influence on NS.SCR frequency & SCL |  |
|  | age + ambient temperature + gender + medication + smoking status + BMI + health status (CCI-score) + | Covariates with protentional influence on HR, HRV & RR |  |
|  | age + medication + | Covariates with protentional influence on BCPD |  |
| assessment phase × predictor + | | Interaction effect |  |
| (1 \| participant) | |  | Random term |

**Supplementary table 6. Main and interaction effect analysis for physiological craving parameters**

**Model formula:** see supplementary table 5.

**Model Output**

| Predictor class | Predictor | Physiological craving parameter | Main effect analysis | |  | Interaction effect analysis | | |
| --- | --- | --- | --- | --- | --- | --- | --- | --- |
|  |  |  | Regression analysis | |  | Regression analysis | | |
|  |  |  | Regression | p-Value |  | Regression | p-Value | ΔR² |
| VR characteristics | Type of risk scenario | RR | F(2, 1259.19) = 4.15 | .016 |  | F(20, 1225.02) = .26 | 1 |  |
|  |  | NS.SCR | F(2, 1227.5) = 1.6 | .202 |  | F(20, 1183.05) = 0.50 | .969 |  |
|  |  | SCL | F(2, 1246.11) = 5.64 | .004 |  | F(20, 1225) = 0.19 | 1 |  |
|  |  | BCPD | F(2, 1231.75) = 0.12 | .886 |  | F(20, 1184.11) = 0.98 | .486 |  |
|  |  | HR | F(2, 1240.93) = 13.8 | <.001 |  | F(20, 1214.02) = 1.48 | .080 |  |
|  | Drink choice | RR | F(4, 40.04) = 0.86 | .495 |  | F(40, 1205.02) = 0.73 | .890 |  |
|  |  | NS.SCR | F(4, 41.11) = 0.19 | .941 |  | F(40, 1163.04) = 0.57 | .986 |  |
|  |  | SCL | F(4, 43) = 0.5 | .738 |  | F(40, 1205) = 0.23 | 1 |  |
|  |  | BCPD | F(4, 44.22) = 0.55 | .697 |  | F(40, 1164.11) = 0.89 | .673 |  |
|  |  | HR | F(4, 40.02) = 2.81 | .038 |  | F(40, 1194.02) = 0.74 | .879 |  |
|  | Order of the exposure | RR | F(1, 1248.9) = 4.44 | .035 |  | F(10, 1235.02) = 1.06 | .390 |  |
|  |  | NS.SCR | F(1, 1210.74) = 15.61 | <.001 |  | F(10, 1193.04) = 1.15 | .320 |  |
|  |  | SCL | F(1, 1245.28) = 170.56 | <.001 |  | F(10, 1235) = 0.19 | .997 |  |
|  |  | BCPD | F(1, 1216.61) = 7.21 | .007 |  | F(10, 1194.11) = 0.18 | .998 |  |
|  |  | HR | F(1, 1236.26) = 229.44 | <.001 |  | F(10, 1224.02) = 0.98 | .460 |  |
| AUD characteristics | AUD identification score (AUDIT) | RR | F(1, 39.99) = 0.21 | .650 |  | F(10, 1235.02) = 2.42 | .007 | 0.007 |
|  |  | NS.SCR | F(1, 40.99) = 0.07 | .788 |  | F(10, 1193.04) = 0.95 | .486 |  |
|  |  | SCL | F(1, 43) = 2.78 | .103 |  | F(10, 1235) = 0.26 | .990 |  |
|  |  | BCPD | F(1, 44.05) = 5.3 | .026 |  | F(10, 1194.11) = 0.50 | .892 |  |
|  |  | HR | F(1, 40.02) = 5.29 | .027 |  | F(10, 1224.02) = 0.28 | .986 |  |
|  | AUD severity score (ADS) | RR | F(1, 39.99) = 1.13 | .294 |  | F(10, 1235.02) = 2.65 | .003 | 0.008 |
|  |  | NS.SCR | F(1, 40.99) = 6.87 | .012 |  | F(10, 1193.04) = 0.14 | .999 |  |
|  |  | SCL | F(1, 43) = 0.21 | .648 |  | F(10, 1235) = 0.17 | .998 |  |
|  |  | BCPD | F(1, 44.03) = 1.5 | .227 |  | F(10, 1194.11) = 0.82 | .608 |  |
|  |  | HR | F(1, 40.12) = 1.93 | .173 |  | F(10, 1224.02) = 0.27 | .988 |  |
|  | Amount of alcohol consumed in the past five years | RR | F(1, 40) = 0.59 | .446 |  | F(10, 1235.02) = 0.87 | .562 |  |
|  |  | NS.SCR | F(1, 40.99) = 2.07 | .158 |  | F(10, 1193.04) = 0.38 | .955 |  |
|  |  | SCL | F(1, 43) = 0.32 | .574 |  | F(10, 1235) = 0.38 | .956 |  |
|  |  | BCPD | F(1, 44.09) = 0.32 | .573 |  | F(10, 1194.11) = 0.68 | .746 |  |
|  |  | HR | F(1, 40) = 0.04 | .840 |  | F(10, 1224.02) = 0.24 | .992 |  |
|  | Days since last alcohol consumption | RR | F(1, 39.99) = 0.09 | .767 |  | F(10, 1235.02) = 2.17 | .018 | 0.006 |
|  |  | NS.SCR | F(1, 40.99) = 0.09 | .770 |  | F(10, 1193.04) = 0.52 | .875 |  |
|  |  | SCL | F(1, 43) = 0.42 | .518 |  | F(10, 1235) = 0.05 | 1 |  |
|  |  | BCPD | F(1, 43.99) = 0.1 | .753 |  | F(10, 1194.11) = 0.26 | .989 |  |
|  |  | HR | F(1, 40.01) = 14.48 | <.001 |  | F(10, 1224.02) = 0.33 | .974 |  |
| Pre-VR craving characteristics | AUQ score | RR | F(1, 40.04) = 0.01 | .918 |  | F(10, 1235.02) = 0.94 | .494 |  |
|  |  | NS.SCR | F(1, 41.22) = 6.06 | .018 |  | F(10, 1193.04) = 0.43 | .930 |  |
|  |  | SCL | F(1, 43) = 0.66 | .422 |  | F(10, 1235) = 0.13 | .999 |  |
|  |  | BCPD | F(1, 44.13) = 0.99 | .325 |  | F(10, 1194.11) = 1.45 | .154 |  |
|  |  | HR | F(1, 40.03) = 1.14 | .292 |  | F(10, 1224.02) = 0.16 | .998 |  |
|  | OCDS score | RR | F(1, 40.06) = 1.53 | .223 |  | F(10, 1235.02) = 2.21 | .015 | 0.007 |
|  |  | NS.SCR | F(1, 41.16) = 7.78 | .008 |  | F(10, 1193.04) = 0.42 | .937 |  |
|  |  | SCL | F(1, 43) = 0.29 | .591 |  | F(10, 1235) = 0.18 | .998 |  |
|  |  | BCPD | F(1, 44.17) = 1.67 | .203 |  | F(10, 1194.11) = 0.45 | .920 |  |
|  |  | HR | F(1, 40.04) = 1.12 | .297 |  | F(10, 1224.02) = 0.64 | .782 |  |
|  | CAS-A g-factor 1 („unaware“) score | RR | F(1, 39.99) = 0 | .953 |  | F(10, 1235.02) = 1.21 | .282 |  |
|  |  | NS.SCR | F(1, 40.98) = 1.01 | .322 |  | F(10, 1193.04) = 0.17 | .998 |  |
|  |  | SCL | F(1, 43) = 1.96 | .169 |  | F(10, 1235) = 0.15 | .999 |  |
|  |  | BCPD | F(1, 44.08) = 0.01 | .920 |  | F(10, 1194.11) = 0.34 | .969 |  |
|  |  | HR | F(1, 40) = 6.63 | .014 |  | F(10, 1224.02) = 0.82 | .611 |  |
|  | CAS-A g-factor 2 („non-volitional“) score | RR | F(1, 39.99) = 1.29 | 0.264 |  | F(10, 1235.03) = 0.93 | .507 |  |
|  |  | NS.SCR | F(1, 40.99) = 1.67 | 0.204 |  | F(10, 1193.03) = 0.45 | .921 |  |
|  |  | SCL | F(1, 43) = 0.47 | 0.495 |  | F(10, 1235) = 0.47 | .913 |  |
|  |  | BCPD | F(1, 44.31) = 0.24 | 0.624 |  | F(10, 1194.13) = 0.32 | .977 |  |
|  |  | HR | F(1, 40.01) = 3.82 | 0.058 |  | F(10, 1224.01) = 0.15 | .999 |  |

**Supplementary Table 7. Main and interaction effect analysis for HRV parameters**

**Model formula:** see supplementary table 5.

**Model Output**

|  |  |  | Main effect analysis | |  | Interaction effect analysis | | |
| --- | --- | --- | --- | --- | --- | --- | --- | --- |
| Predictor class | Predictor | HRV craving parameter | Regression analysis | |  | Regression analysis | | |
|  |  |  | Regression | p-Value |  | Regression | p-Value | ΔR² |
| VR characteristics | Type of risk scenario | HFB | F(2, 214) = 1.75 | .175 |  | F(2, 212) = 2.15 | .119 |  |
|  |  | HF/(LF+HF) | F(2, 191.58) = 1.84 | .161 |  | F(2, 172.37) = 0.07 | .937 |  |
|  |  | LFB | F(2, 209.58) = 0.96 | .383 |  | F(2, 172.69) = 0.74 | .478 |  |
|  |  | LF/HF | F(2, 197.11) = 0.25 | .775 |  | F(2, 172.52) = 0.28 | .755 |  |
|  |  | LF/(LF+HF) | F(2, 191.58) = 1.84 | .161 |  | F(2, 172.37) = 0.07 | .937 |  |
|  |  | pNN50 | F(2, 179.34) = 3.22 | .042 |  | F(2, 172.09) = 1.11 | .333 |  |
|  |  | RMSSD | F(2, 191.49) = 2.52 | .083 |  | F(2, 172.37) = 0.30 | .742 |  |
|  |  | SD1 | F(2, 210.07) = 0.45 | .639 |  | F(2, 173.17) = 0.40 | .669 |  |
|  |  | SD1/SD2 | F(2, 190.28) = 4.33 | .014 |  | F(2, 172.42) = 0.27 | .761 |  |
|  |  | SD2 | F(2, 186.76) = 0.74 | .478 |  | F(2, 172.21) = 1.25 | .290 |  |
|  |  | SDRR | F(2, 209.25) = 1.75 | .176 |  | F(2, 172.91) = 2.21 | .113 |  |
|  |  | SDSD | F(2, 191.49) = 2.52 | .083 |  | F(2, 172.37) = 0.30 | .742 |  |
|  |  | total power | F(2, Inf) = 1.74 | .175 |  | F(2, Inf) = 2.28 | .102 |  |
|  |  | VHFB | F(2, 214) = 1.64 | .196 |  | F(2, 212) = 2.16 | .118 |  |
|  |  | VLFB | F(2, 197.86) = 0.56 | .574 |  | F(2, 171.65) = 5.47 | .005 | 0.020 |
|  | Drink choice | HFB | F(4, 214) = 0.25 | .912 |  | F(4, 210) = 1.27 | .285 |  |
|  |  | HF/(LF+HF) | F(4, 40.39) = 0.52 | .725 |  | F(4, 170.37) = 0.79 | .531 |  |
|  |  | LFB | F(4, 40.56) = 1.3 | .286 |  | F(4, 170.68) = 1.65 | .164 |  |
|  |  | LF/HF | F(4, 40.52) = 0.77 | .549 |  | F(4, 170.52) = 0.29 | .883 |  |
|  |  | LF/(LF+HF) | F(4, 40.39) = 0.52 | .725 |  | F(4, 170.37) = 0.79 | .531 |  |
|  |  | pNN50 | F(4, 40.11) = 2.01 | .111 |  | F(4, 170.1) = 0.41 | .799 |  |
|  |  | RMSSD | F(4, 40.39) = 1.68 | .173 |  | F(4, 170.37) = 0.51 | .728 |  |
|  |  | SD1 | F(4, 40.91) = 0.22 | .925 |  | F(4, 171.17) = 0.45 | .775 |  |
|  |  | SD1/SD2 | F(4, 40.44) = 0.73 | .575 |  | F(4, 170.41) = 1.05 | .382 |  |
|  |  | SD2 | F(4, 40.24) = 2.82 | .037 |  | F(4, 170.22) = 0.15 | .964 |  |
|  |  | SDRR | F(4, 40.8) = 3.26 | .021 |  | F(4, 170.91) = 1.42 | .231 |  |
|  |  | SDSD | F(4, 40.39) = 1.68 | .173 |  | F(4, 170.37) = 0.51 | .728 |  |
|  |  | total power | F(4, Inf) = 0.25 | .908 |  | F(4, Inf) = 1.35 | .247 |  |
|  |  | VHFB | F(4, 214) = 0.24 | .916 |  | F(4, 210) = 1.27 | .282 |  |
|  |  | VLFB | F(4, 39.64) = 1.25 | .306 |  | F(4, 169.64) = 0.84 | .504 |  |
|  | Order of the exposure | HFB | F(1, 214) = 1.65 | .200 |  | F(1, 213) = 1.05 | .307 |  |
|  |  | HF/(LF+HF) | F(1, 179.37) = 7.86 | .006 |  | F(1, 173.37) = 0.44 | .508 |  |
|  |  | LFB | F(1, 184.3) = 0.99 | .321 |  | F(1, 173.69) = 1.50 | .223 |  |
|  |  | LF/HF | F(1, 180.91) = 3.95 | .048 |  | F(1, 173.52) = 0.78 | .380 |  |
|  |  | LF/(LF+HF) | F(1, 179.37) = 7.86 | .006 |  | F(1, 173.37) = 0.44 | .508 |  |
|  |  | pNN50 | F(1, 175.7) = 21.84 | <.001 |  | F(1, 173.09) = 1.13 | .289 |  |
|  |  | RMSSD | F(1, 179.34) = 19.84 | <.001 |  | F(1, 173.37) = 0.21 | .647 |  |
|  |  | SD1 | F(1, 185.84) = 1.89 | .171 |  | F(1, 174.16) = 0.93 | .337 |  |
|  |  | SD1/SD2 | F(1, 179.05) = 12 | <.001 |  | F(1, 173.42) = 0.00 | .994 |  |
|  |  | SD2 | F(1, 177.94) = 30 | <.001 |  | F(1, 173.21) = 0.18 | 0.674 |  |
|  |  | SDRR | F(1, 184.36) = 8.38 | .004 |  | F(1, 173.92) = 0.47 | 0.495 |  |
|  |  | SDSD | F(1, 179.34) = 19.83 | <.001 |  | F(1, 173.37) = 0.21 | 0.647 |  |
|  |  | total power | F(1, 1.37693037361975e+40) = 1.79 | .181 |  | F(1, 2.83938845231439e+40) = 0.99 | 0.320 |  |
|  |  | VHFB | F(1, 214) = 1.96 | .163 |  | F(1, 213) = 1.04 | 0.309 |  |
|  |  | VLFB | F(1, 180.45) = 7.66 | .006 |  | F(1, 172.64) = 0.03 | 0.856 |  |
| AUD characteristics | AUD identification score (AUDIT) | HFB | F(1, 214) = 0.02 | .899 |  | F(1, 213) = 0.01 | 0.907 |  |
|  |  | HF/(LF+HF) | F(1, 40.02) = 0.29 | .594 |  | F(1, 173.36) = 3.60 | 0.060 |  |
|  |  | LFB | F(1, 39.77) = 0.02 | .881 |  | F(1, 173.69) = 0.43 | 0.515 |  |
|  |  | LF/HF | F(1, 40.03) = 0.06 | .812 |  | F(1, 173.51) = 2.48 | 0.117 |  |
|  |  | LF/(LF+HF) | F(1, 40.02) = 0.29 | .594 |  | F(1, 173.36) = 3.60 | .060 |  |
|  |  | pNN50 | F(1, 40) = 0.19 | .667 |  | F(1, 173.09) = 0.00 | .955 |  |
|  |  | RMSSD | F(1, 40.02) = 0.91 | .345 |  | F(1, 173.37) = 0.98 | .323 |  |
|  |  | SD1 | F(1, 39.99) = 0.05 | .817 |  | F(1, 174.16) = 0.85 | .357 |  |
|  |  | SD1/SD2 | F(1, 40.09) = 0.03 | .859 |  | F(1, 173.41) = 2.13 | .146 |  |
|  |  | SD2 | F(1, 39.97) = 0.01 | .904 |  | F(1, 173.21) = 0.76 | .383 |  |
|  |  | SDRR | F(1, 40.02) = 1.43 | .239 |  | F(1, 173.92) = 0.70 | .405 |  |
|  |  | SDSD | F(1, 40.02) = 0.91 | .345 |  | F(1, 173.37) = 0.98 | .323 |  |
|  |  | total power | F(1, 1.37693037362393e+40) = 0.08 | .772 |  | F(1, 3.38639623736978e+39) = 0.02 | .892 |  |
|  |  | VHFB | F(1, 214) = 0.06 | .810 |  | F(1, 213) = 0.01 | .910 |  |
|  |  | VLFB | F(1, 39.13) = 0.01 | .942 |  | F(1, 172.64) = 0.07 | .798 |  |
|  | AUD severity score (ADS) | HFB | F(1, 214) = 0.85 | .358 |  | F(1, 213) = 1.19 | .276 |  |
|  |  | HF/(LF+HF) | F(1, 39.89) = 0.01 | .920 |  | F(1, 173.37) = 0.83 | .364 |  |
|  |  | LFB | F(1, 39.55) = 0.32 | .576 |  | F(1, 173.68) = 2.20 | .140 |  |
|  |  | LF/HF | F(1, 39.88) = 0.15 | .703 |  | F(1, 173.52) = 1.06 | .305 |  |
|  |  | LF/(LF+HF) | F(1, 39.89) = 0.01 | .920 |  | F(1, 173.37) = 0.83 | .364 |  |
|  |  | pNN50 | F(1, 39.96) = 1.48 | .231 |  | F(1, 173.09) = 0.66 | .419 |  |
|  |  | RMSSD | F(1, 39.9) = 1.03 | .316 |  | F(1, 173.37) = 0.26 | .608 |  |
|  |  | SD1 | F(1, 39.76) = 0.28 | .601 |  | F(1, 174.16) = 0.37 | .545 |  |
|  |  | SD1/SD2 | F(1, 39.98) = 0.74 | .395 |  | F(1, 173.42) = 0.04 | .846 |  |
|  |  | SD2 | F(1, 39.87) = 2.94 | .094 |  | F(1, 173.21) = 1.57 | .212 |  |
|  |  | SDRR | F(1, 39.81) = 1.43 | .238 |  | F(1, 173.92) = 1.18 | .279 |  |
|  |  | SDSD | F(1, 39.9) = 1.03 | .316 |  | F(1, 173.37) = 0.26 | .608 |  |
|  |  | total power | F(1, 1.37693037360915e+40) = 1.01 | .316 |  | F(1, 1.22438127694909e+40) = 1.24 | .266 |  |
|  |  | VHFB | F(1, 214) = 1.07 | .303 |  | F(1, 213) = 1.19 | .278 |  |
|  |  | VLFB | F(1, 38.98) = 0.43 | .514 |  | F(1, 172.64) = 0.05 | .817 |  |
|  | Amount of alcohol consumed in the past five years | HFB | F(1, 214) = 0.02 | .880 |  | F(1, 213) = 0.36 | .547 |  |
|  |  | HF/(LF+HF) | F(1, 39.99) = 1.47 | .232 |  | F(1, 173.37) = 0.59 | .442 |  |
|  |  | LFB | F(1, 39.75) = 0.08 | .776 |  | F(1, 173.69) = 1.29 | .258 |  |
|  |  | LF/HF | F(1, 40) = 0.03 | .867 |  | F(1, 173.52) = 1.02 | .314 |  |
|  |  | LF/(LF+HF) | F(1, 39.99) = 1.47 | .232 |  | F(1, 173.37) = 0.59 | .442 |  |
|  |  | pNN50 | F(1, 39.99) = 0.34 | .561 |  | F(1, 173.09) = 1.04 | .309 |  |
|  |  | RMSSD | F(1, 39.99) = 0.65 | .425 |  | F(1, 173.37) = 0.40 | .528 |  |
|  |  | SD1 | F(1, 39.99) = 0 | .956 |  | F(1, 174.16) = 0.11 | .737 |  |
|  |  | SD1/SD2 | F(1, 40.07) = 0.2 | .656 |  | F(1, 173.41) = 0.99 | .320 |  |
|  |  | SD2 | F(1, 39.95) = 1.33 | .255 |  | F(1, 173.21) = 0.41 | .525 |  |
|  |  | SDRR | F(1, 40.01) = 1.65 | .207 |  | F(1, 173.92) = 0.65 | .423 |  |
|  |  | SDSD | F(1, 39.99) = 0.65 | .425 |  | F(1, 173.37) = 0.40 | .528 |  |
|  |  | total power | F(1, 1.37693037360931e+40) = 0.03 | .871 |  | F(1, 6.57677666233235e+39) = 0.41 | .523 |  |
|  |  | VHFB | F(1, 214) = 0.04 | .845 |  | F(1, 213) = 0.37 | .542 |  |
|  |  | VLFB | F(1, 39.11) = 0.65 | .427 |  | F(1, 172.64) = 0.48 | .487 |  |
|  | Days since last alcohol consumption | HFB | F(1, 214) = 0.14 | .707 |  | F(1, 213) = 0.25 | .620 |  |
|  |  | HF/(LF+HF) | F(1, 40.04) = 3.06 | .088 |  | F(1, 173.37) = 0.25 | .616 |  |
|  |  | LFB | F(1, 39.83) = 0.31 | .578 |  | F(1, 173.69) = 0.26 | .612 |  |
|  |  | LF/HF | F(1, 40.07) = 1.19 | .282 |  | F(1, 173.52) = 0.07 | .786 |  |
|  |  | LF/(LF+HF) | F(1, 40.04) = 3.06 | .088 |  | F(1, 173.37) = 0.25 | .616 |  |
|  |  | pNN50 | F(1, 40.01) = 6.15 | .017 |  | F(1, 173.09) = 0.06 | .813 |  |
|  |  | RMSSD | F(1, 40.05) = 8.3 | .006 |  | F(1, 173.37) = 0.44 | .510 |  |
|  |  | SD1 | F(1, 40.07) = 0.18 | .671 |  | F(1, 174.16) = 0.24 | .624 |  |
|  |  | SD1/SD2 | F(1, 40.12) = 0.42 | .519 |  | F(1, 173.42) = 0.16 | .693 |  |
|  |  | SD2 | F(1, 39.99) = 6.23 | .017 |  | F(1, 173.21) = 0.28 | .599 |  |
|  |  | SDRR | F(1, 40.09) = 5.33 | .026 |  | F(1, 173.92) = 0.43 | .513 |  |
|  |  | SDSD | F(1, 40.05) = 8.3 | .006 |  | F(1, 173.37) = 0.44 | .510 |  |
|  |  | total power | F(1, 1.37693037360268e+40) = 0.05 | .818 |  | F(1, 4.4631630048269e+39) = 0.26 | .613 |  |
|  |  | VHFB | F(1, 214) = 0.03 | .857 |  | F(1, 213) = 0.28 | .598 |  |
|  |  | VLFB | F(1, 39.17) = 3.12 | .085 |  | F(1, 172.65) = 3.12 | .079 |  |
| Pre-VR craving characteristics | AUQ score | HFB | F(1, 214) = 0.58 | .447 |  | F(1, 213) = 0.76 | .383 |  |
|  |  | HF/(LF+HF) | F(1, 40.27) = 2.07 | .157 |  | F(1, 173.37) = 0.14 | .710 |  |
|  |  | LFB | F(1, 40.33) = 0.01 | .904 |  | F(1, 173.68) = 1.64 | .202 |  |
|  |  | LF/HF | F(1, 40.37) = 2.3 | .137 |  | F(1, 173.52) = 0.06 | .813 |  |
|  |  | LF/(LF+HF) | F(1, 40.27) = 2.07 | .157 |  | F(1, 173.37) = 0.14 | .710 |  |
|  |  | pNN50 | F(1, 40.07) = 2.44 | .126 |  | F(1, 173.09) = 1.03 | .311 |  |
|  |  | RMSSD | F(1, 40.27) = 0.36 | .551 |  | F(1, 173.37) = 0.94 | .335 |  |
|  |  | SD1 | F(1, 40.66) = 2.1 | .155 |  | F(1, 174.17) = 0.01 | .930 |  |
|  |  | SD1/SD2 | F(1, 40.32) = 0.35 | .556 |  | F(1, 173.41) = 1.29 | .257 |  |
|  |  | SD2 | F(1, 40.15) = 0.02 | .900 |  | F(1, 173.21) = 1.00 | .320 |  |
|  |  | SDRR | F(1, 40.58) = 0.01 | .939 |  | F(1, 173.92) = 0.03 | .865 |  |
|  |  | SDSD | F(1, 40.27) = 0.36 | .551 |  | F(1, 173.37) = 0.94 | .335 |  |
|  |  | total power | F(1, 1.37693037361508e+40) = 0.54 | .462 |  | F(1, 1.90516110547442e+38) = 0.75 | .387 |  |
|  |  | VHFB | F(1, 214) = 0.55 | .461 |  | F(1, 213) = 0.74 | .392 |  |
|  |  | VLFB | F(1, 39.48) = 0.7 | .407 |  | F(1, 172.64) = 0.43 | .515 |  |
|  | OCDS score | HFB | F(1, 214) = 0.29 | .592 |  | F(1, 213) = 0.01 | .931 |  |
|  |  | HF/(LF+HF) | F(1, 40.54) = 0.58 | .452 |  | F(1, 173.37) = 3.14 | .078 |  |
|  |  | LFB | F(1, 40.9) = 0.39 | .536 |  | F(1, 173.69) = 0.33 | .564 |  |
|  |  | LF/HF | F(1, 40.72) = 0.02 | .883 |  | F(1, 173.52) = 0.07 | .787 |  |
|  |  | LF/(LF+HF) | F(1, 40.54) = 0.58 | .452 |  | F(1, 173.37) = 3.14 | .078 |  |
|  |  | pNN50 | F(1, 40.15) = 0.98 | .328 |  | F(1, 173.09) = 1.46 | .228 |  |
|  |  | RMSSD | F(1, 40.54) = 1.59 | .214 |  | F(1, 173.37) = 0.89 | .346 |  |
|  |  | SD1 | F(1, 41.33) = 2.53 | .119 |  | F(1, 174.16) = 2.12 | .147 |  |
|  |  | SD1/SD2 | F(1, 40.57) = 0.99 | .325 |  | F(1, 173.41) = 1.30 | .256 |  |
|  |  | SD2 | F(1, 40.34) = 3.26 | .079 |  | F(1, 173.21) = 0.02 | .897 |  |
|  |  | SDRR | F(1, 41.15) = 6.95 | .012 |  | F(1, 173.92) = 0.40 | .529 |  |
|  |  | SDSD | F(1, 40.54) = 1.59 | .214 |  | F(1, 173.37) = 0.89 | .346 |  |
|  |  | total power | F(1, 1.37693037361013e+40) = 0.26 | .613 |  | F(1, 2.94605001476569e+39) = 0.02 | .900 |  |
|  |  | VHFB | F(1, 214) = 0.34 | .563 |  | F(1, 213) = 0.01 | .925 |  |
|  |  | VLFB | F(1, 39.85) = 0.37 | .546 |  | F(1, 172.65) = 1.91 | .169 |  |
|  | CAS-A g-factor 1 („unaware“) score | HFB | F(1, 214) = 0.03 | .865 |  | F(1, 213) = 0.11 | .736 |  |
|  |  | HF/(LF+HF) | F(1, 39.89) = 0.31 | .581 |  | F(1, 173.37) = 1.07 | .303 |  |
|  |  | LFB | F(1, 39.53) = 0.04 | .841 |  | F(1, 173.69) = 0.69 | .408 |  |
|  |  | LF/HF | F(1, 39.86) = 2.32 | .135 |  | F(1, 173.51) = 2.70 | .102 |  |
|  |  | LF/(LF+HF) | F(1, 39.89) = 0.31 | .581 |  | F(1, 173.37) = 1.07 | .303 |  |
|  |  | pNN50 | F(1, 39.95) = 3.86 | .057 |  | F(1, 173.09) = 0.35 | .557 |  |
|  |  | RMSSD | F(1, 39.89) = 0.78 | .381 |  | F(1, 173.37) = 0.14 | .711 |  |
|  |  | SD1 | F(1, 39.74) = 0.72 | .401 |  | F(1, 174.16) = 0.31 | .581 |  |
|  |  | SD1/SD2 | F(1, 39.97) = 0.01 | .938 |  | F(1, 173.42) = 0.00 | .978 |  |
|  |  | SD2 | F(1, 39.87) = 3.07 | .087 |  | F(1, 173.21) = 0.25 | .618 |  |
|  |  | SDRR | F(1, 39.79) = 6.4 | .016 |  | F(1, 173.92) = 1.63 | .203 |  |
|  |  | SDSD | F(1, 39.89) = 0.78 | .381 |  | F(1, 173.37) = 0.14 | .711 |  |
|  |  | total power | F(1, 1.37693037362518e+40) = 0.01 | .913 |  | F(1, 3.34608037054786e+39) = 0.10 | .750 |  |
|  |  | VHFB | F(1, 214) = 0.03 | .869 |  | F(1, 213) = 0.14 | .710 |  |
|  |  | VLFB | F(1, 38.97) = 0.85 | .361 |  | F(1, 172.64) = 1.03 | .312 |  |
|  | CAS-A g-factor 2 („non-volitional“) score | HFB | F(1, 214) = 0.02 | .887 |  | F(1, 174.26) = 0.39 | .531 |  |
|  |  | HF/(LF+HF) | F(1, 40) = 2.12 | .153 |  | F(1, 173.41) = 1.66 | .200 |  |
|  |  | LFB | F(1, 39.74) = 2.5 | .121 |  | F(1, 173.61) = 0.01 | .942 |  |
|  |  | LF/HF | F(1, 40.01) = 2.44 | .126 |  | F(1, 173.57) = 2.88 | .091 |  |
|  |  | LF/(LF+HF) | F(1, 40) = 2.12 | .153 |  | F(1, 173.41) = 1.66 | .200 |  |
|  |  | pNN50 | F(1, 39.99) = 1.22 | .277 |  | F(1, 173.04) = 0.02 | .885 |  |
|  |  | RMSSD | F(1, 40) = 0.02 | .885 |  | F(1, 173.36) = 0.08 | .773 |  |
|  |  | SD1 | F(1, 39.97) = 0.02 | .881 |  | F(1, 174.26) = 0.02 | .877 |  |
|  |  | SD1/SD2 | F(1, 40.07) = 0.92 | .342 |  | F(1, 173.38) = 1.33 | .251 |  |
|  |  | SD2 | F(1, 39.95) = 0.07 | .794 |  | F(1, 173.04) = 0.30 | .588 |  |
|  |  | SDRR | F(1, 40) = 0.47 | .496 |  | F(1, 173.51) = 0.00 | .951 |  |
|  |  | SDSD | F(1, 40) = 0.02 | .885 |  | F(1, 173.36) = 0.08 | .773 |  |
|  |  | total power | F(1, 1.3769303736191e+40) = 0.01 | .943 |  | F(1, 5.27744029931253e+20) = 0.41 | .523 |  |
|  |  | VHFB | F(1, 214) = 0.01 | .934 |  | F(1, 174.27) = 0.42 | .516 |  |
|  |  | VLFB | F(1, 39.11) = 0.02 | .893 |  | F(1, 172.5) = 2.27 | .133 |  |

**Supplementary Table 8. Post hoc analysis of significant interaction effects for HRV parameter VLFB during VR-CE**

**Model formula:** see supplementary table 5.

**Model Output**

| HRV Parameter | Predictor class | Post hoc analysis of significant interaction effects | | | | |
| --- | --- | --- | --- | --- | --- | --- |
|  |  |  |  |  | Interaction contrast | |
|  |  | Assessment × predictor interaction | Interaction | Contrasts^1^ | β (95% CI) | p-Value |
| VLFB | VR characteristics | Type of risk scenario | ×R | Living room | 16.02 (-1.83, 33.88) | .063 |
|  |  |  |  | Pub | -24.89 (-44.78, -4.99) | .009 |
|  |  |  |  | Wine bar | 8.86 (-13.6, 31.33) | .341 |
| Contrasts of the interaction effect of two categorical predictors using effect coding. | | | | | | |

**Supplementary Table 9. Post hoc analysis of significant interaction effects for RR during VR-CE**

**Model formula:** see supplementary table 5.

**Model Output**

| Predictor class |  | Post hoc analysis of significant interaction effects | | | | | | |
| --- | --- | --- | --- | --- | --- | --- | --- | --- |
|  |  |  | Interaction contrasts | |  |  | Simple slope analysis | |
|  | Assessment × predictor interaction | Interaction | β (95% CI) | p-Value |  |  | β (95% CI) | p-Value |
| AUD characteristics | AUD screening score (AUDIT) | ×R4 | -0.79 (-1.39, -0.19) | .010 |  | -1 SD | 1.46 (0.62, 2.31) | .001 |
|  |  |  |  |  |  | Mean | 0.67 (0.07, 1.27) | .028 |
|  |  |  |  |  |  | +1 SD | -0.12 (-0.97, 0.73) | .786 |
|  | AUD severity score (ADS) | ×R3 | -0.61 (-1.21, -0.01) | .045 |  | -1 SD | 1.39 (0.55, 2.24) | .001 |
|  |  |  |  |  |  | Mean | 0.78 (0.18, 1.38) | .011 |
|  |  |  |  |  |  | +1 SD | 0.17 (-0.68, 1.01) | .699 |
|  |  | ×R4 | -0.71 (-1.31, -0.11) | .020 |  | -1 SD | 1.38 (0.54, 2.23) | .001 |
|  |  |  |  |  |  | Mean | 0.67 (0.07, 1.27) | .028 |
|  |  |  |  |  |  | +1 SD | -0.04 (-0.88, 0.81) | .934 |
|  |  | ×R5 | -0.72 (-1.31, -0.12) | .019 |  | -1 SD | 1.73 (0.89, 2.58) | <.001 |
|  |  |  |  |  |  | Mean | 1.02 (0.42, 1.62) | .001 |
|  |  |  |  |  |  | +1 SD | 0.3 (-0.55, 1.15) | .487 |
|  | Days since last alcohol consumption | ×R1 | 0.63 (0.03, 1.23) | .041 |  | -1 SD | 1.03 (0.18, 1.88) | .017 |
|  |  |  |  |  |  | Mean | 1.66 (1.06, 2.26) | <.001 |
|  |  |  |  |  |  | +1 SD | 2.29 (1.44, 3.13) | <.001 |
| Pre-VR craving characteristics | OCDS score | ×R1 | -0.81 (-1.41, -0.21) | .008 |  | -1 SD | 2.47 (1.62, 3.31) | <.001 |
|  |  |  |  |  |  | Mean | 1.66 (1.06, 2.26) | <.001 |
|  |  |  |  |  |  | +1 SD | 0.85 (0, 1.7) | .049 |

**Suppl. Figure 1**. Linear mixed-effect models displaying interaction effects of *AUDIT score* × VR-CE assessments at R5 on subjective craving measured by visual analogue scale (VAS). Lines represent model-estimated simple slopes at low (−1 SD), mean, and high (+1 SD) levels of *AUDIT score*. Error bars indicate 95% confidence intervals.

**Suppl. Figure 2.** Linear mixed-effect models displaying interaction effects of *ADS score* × VR-CE assessments at R3 (a) and R5 (b) on subjective craving measured by visual analogue scale (VAS). Lines represent model-estimated simple slopes at low (−1 SD), mean, and high (+1 SD) levels of *ADS score*. Error bars indicate 95% confidence intervals.

**Suppl. Figure 3**. Linear mixed-effect models displaying interaction effects of *amount of* *alcohol consumed in the past five years* (pure alcohol, in kilogram) × VR-CE assessments at R3 (a) and R5 (b) on subjective craving measured by visual analogue scale (VAS). Lines represent model-estimated simple slopes at low (−1 SD), mean, and high (+1 SD) levels of *amount of* *alcohol consumed in the past five years* (pure alcohol, in kilogram). Error bars indicate 95% confidence intervals.

**Suppl. Figure 4**. Linear mixed-effect models displaying interaction effects of *AUQ score* × VR-CE assessments at R3 (a) and R5 (b) on subjective craving measured by visual analogue scale (VAS). Lines represent model-estimated simple slopes at low (−1 SD), mean, and high (+1 SD) levels of *AUQ score*. Error bars indicate 95% confidence intervals.

**Suppl. Figure 5.** Linear mixed-effect models displaying interaction effects of *AUDIT score* × VR-CE assessments at R4 on respiration rate in breaths per minute (BPM). Lines represent model-estimated simple slopes at low (−1 SD), mean, and high (+1 SD) levels of *AUDIT score*. Error bars indicate 95% confidence intervals.

**Suppl. Figure 6.** Linear mixed-effect models displaying interaction effects of *ADS score* × VR-CE assessments at R3 (a), R4 (b) and R5 (c) on respiration rate in breaths per minute (BPM). Lines represent model-estimated simple slopes at low (−1 SD), mean, and high (+1 SD) levels of *ADS score*. Error bars indicate 95% confidence intervals.

**Suppl. Figure 7**. Linear mixed-effect models displaying interaction effects of *days since last alcohol consumption*× VR-CE assessments at R1 on respiration rate in breaths per minute (BPM). Lines represent model-estimated simple slopes at low (−1 SD), mean, and high (+1 SD) levels of *days since last alcohol consumption*. Error bars indicate 95% confidence intervals.

**Suppl. Figure 8.** Linear mixed-effect models displaying interaction effects of *OCDS score* × VR-CE assessments at R1 on respiration rate in breaths per minute (BPM). Lines represent model-estimated simple slopes at low (−1 SD), mean, and high (+1 SD) levels of *OCDS score*. Error bars indicate 95% confidence intervals.

**References**

1.	Lütt A, Ruckser N, Turno A, Tsamitros N, Thormann J, Schulze D, et al. Multimodal Psychophysiological Assessment of Craving in Patients With Alcohol Dependence During Virtual Reality Cue Exposure: Exploratory Single-Arm Clinical Study. JMIR Serious Games. 2026;14:e84156.
